# High fat diet increases the severity of collagen-induced arthritis in mice by altering the gut microbial community

**DOI:** 10.1186/s42358-024-00382-y

**Published:** 2024-05-30

**Authors:** Yang Zhang, Jie Zhang, Yantong Liu, Shuang Ren, Ning Tao, Fanyan Meng, Qi Cao, Ruoshi Liu

**Affiliations:** 1 The First Hospital of China Medical University, Shenyang, 110002 Liaoning China; 2 Liaoning University of Traditional Chinese Medicine, Shenyang, 110001 Liaoning China

**Keywords:** Rheumatoid arthritis, Collagen-induced arthritis, High fat diet, Gut microbiota

## Abstract

**Objectives:**

Research has demonstrated that obesity may be associated with rheumatoid arthritis (RA). In addition, gut microbiota and its metabolites contribute to the occurrence and development of RA and obesity. However, the mechanism by which obesity affects RA remains unclear. In this study, we aimed to investigate whether gut microbiota and their metabolites alter the effects of high fat diet (HFD) on the severity of collagen-induced arthritis (CIA) in mice.

**Methods:**

Briefly, mice were divided into normal group (N), CIA model group (C), HFD group (T), and HFD CIA group (CT). Hematoxylin and Eosin staining(HE) and Safranin O-fast green staining were conducted, and levels of blood lipid and inflammatory cytokines were measured. 16S rDNA sequencing technique and liquid chromatography-mass spectrometry (LC-MS)-based metabolomics were performed to explore changes in the microbiota structure to further reveal the pathomechanism of HFD on CIA.

**Results:**

HFD aggravated the severity of CIA in mice. The CT group had the highest proportion of microbial abundance of *Blautia, Oscillibacter, Ruminiclostridium*-9, and *Lachnospiraceae* UCG 006 at the genus level, but had a lower proportion of Alistipes. Additionally, the fecal metabolic phenotype of the combined CT group shows significant changes, with differential metabolites enriched in 9 metabolic pathways, including primary bile acid biosynthesis, arginine biosynthesis, sphingolipid metabolism, purine metabolism, linoleic acid metabolism, oxytocin signaling pathway, aminoacyl-tRNA biosynthesis, the pentose phosphate pathway, and sphingolipid signaling pathway. Correlation analysis revealed that some of the altered gut microbiota genera were strongly correlated with changes in fecal metabolites, total cholesterol (TC), triglyceride (TG), and inflammatory cytokine levels.

**Conclusions:**

This study shows that HFD may aggravate inflammatory reaction in CIA mice by altering the gut microbiota and metabolic pathways.

**Supplementary Information:**

The online version contains supplementary material available at 10.1186/s42358-024-00382-y.

## Introduction

Rheumatoid arthritis (RA) is a chronic autoimmune inflammatory disease of an unknown cause. The pathological hallmarks of joint synovial membranes in RA patients include inflammatory immune cells infiltration at the joints, synovial hyperplasia, micro-vessels and pannus formation, causing articular cartilage and bone erosion and joint destruction [1–4]. Numerous studies have reported that both genetic and environmental factors contribute to RA development [5]. Environmental factors regulate several mechanisms that lead to RA, including modulation of the microbiome [6]. Gut microbiota metabolites can trigger autoimmune dysfunction in genetically susceptible individuals [7]. Molecular simulations of the microbiome may trigger RA [8, 9]. Previous studies have reported that probiotic supplementation can relieve joint inflammation in patients, indicating the potential role of the microbiome as a treatment option for RA [10].

A study in China found that RA patients showed elevated abundance of active *lactobacillus* [11, 12]. In comparison, other studies found that the genus *Prevotella*, especially *P. copri*, was abundant in early RA [13, 14]. The abundance of *Bacteroides* has been found to be decreased in patients with RA [15, 16]. Widian et al. [17] confirmed that gut ecological disorders trigger collagen-induced arthritis (CIA) through mucosal immune responses. “Prevotella-dominated” RA microbiota increased arthritis severity in mice [18]. Fecal bacteria transplantation can improve the leaky barrier and mucosal inflammation in mice [19, 20]. These observations highlight the potential association between gut microbiota and RA. Research on humans and animals has provided strong evidence that RA and gut microbiota interact [21]. Higher proportion of the prevailing evidence indicates that intestinal microbiota affects the occurrence and development of RA. Moreover, changes in gut microbiota can affect the performance of RA [22, 23]. In patient and mouse models, intestinal microflora influences the progression from preclinical RA to clinical RA [15, 24–26]. Most disorders of gut microbiota develop before visible symptoms of arthritis are detected and continue to evolve along the disease course [17, 27]. Liu et al. reported the number and type of intestinal Lactobacillus in patients with early RA were significantly increased [12], and another study showed that the levels of *Bifidobacteria, B. fragilis*, and *Eubacterium rectale* were significantly decreased [28]. In addition, *Prevotella* is associated with the genotype of RA, that is, high-risk individuals with RA [29]. The enrichment of these microbial communities confirms that RA exhibits distinct microbial characteristics at different stages of the disease [30]. When gut microbiota from RA patients were transplanted into SKG mice, it resulted in more severe arthritis [31] and an increase in TH17 cells in the intestine [18]. Recovery from gut microbiota dysbiosis after RA treatment indicates that controlling the gut microbiota may potentially prevent or treat RA [11, 27]. Therefore, we hypothesized that gut microbiota is strongly associated with the onset of RA.

Accumulating evidence indicates that obesity is a metabolic disease characterized by low-grade chronic inflammation, dyslipidemia, insulin resistance, and adipocyte hypertrophy [32–34]. Studies have demonstrated that obesity may be a risk factor for RA development [35], and is considered to be one of the possible reasons for the increase in RA incidence in recent years [36–38]. In RA patients, the Body mass index (BMI) is positively correlated with C-reactive protein and DAS28 score, highlighting the adverse effects of overweight not only on the disease activity itself, but also on the quality of life of patients [39, 40]. Clinical studies have demonstrated that lipid-lowering drugs can improve arthritis symptoms in RA patients [41]. In animal experiments, it was found that obese CIA mice exhibited higher arthritis index scores and histological scores compared to normal CIA mice [42]. Systematic evaluation and meta-analysis also revealed positive correlations among the BMI, fat mass, and RA risk [43]. This notion is supported by the lower response rate to biologics in obese RA patients [44, 45]. Meanwhile, in RA, a sedentary lifestyle and increased exposure to inflammatory mediators can further cause lipid metabolism disorders and exacerbate RA [46–48]. Therefore, obesity play a role in the onset of RA. In future, we will further explore the mechanism by which obesity exacerbates RA.

Research has suggested that the development and progression of obesity are influenced by the interaction of gut microbiota and host metabolism [49–51]. Studies on HFD have consistently revealed a disruption in the ecological balance of gut microbiota, leading to increased gut permeability and elevated levels of bacterial LPS [52–55]. A major change is the reduction in the dominant *Firmicutes/Bacteroidetes* ratio. Moreover, a systematic review revealed differences in the intestinal microflora between obese individuals and normal weight [56]. For example, Bacteroidetes are less common in obese patients [50]. *Prevotella* gain is related to weight gain [57]. In addition, several types of bacteria such as *Lactobacillus* and *Clostridium* are associated with occurrence of metabolic disorders in obese patients [58–60]. Therefore, changing gut microbiota composition may be an effective method of restoring gut functional integrity and reversing the characteristics of obesity [61–64].

Therefore, both RA and HFD have been shown to cause dysbiosis of gut microbiota [65]. Furthermore, an increase in the abundance of the *Odoribacter* genus in patients with RA has also been associated with obesity [66]. Similarly, study found that obesity increases the abundance of *Prevotella*, which is associated with the onset of RA. Therefore, we hypothesize that changes in gut microbiota induced by obesity can increase the risk of RA. To test this hypothesis, 16S rRNA gene sequencing technology was used to detect the species of gut microbiota in feces. The 16S rRNA gene sequencing can reveal microbial diversity, population structure, and interactions [67]. Microbial metabolites can mediate the effects of gut microbiome on host metabolism. Non-targeted metabolomics is a valuable approach for identifying abnormal metabolites and metabolic pathways associated with complex diseases. It is frequently employed to investigate the intricate relationship between gut microbiota and host metabolic phenotype [68]. Fecal metabolomics offers a comprehensive and detailed *in vivo* metabolic profile by uncovering biomarkers relevant to physiological and pathological processes [69, 70]. This study used a CIA model with a HFD to investigate the effect of HFD on CIA mice. This study also used the 16S rRNA sequence analysis to analyze fecal samples to identify the changed gut microbiota. On the other hand, the liquid chromatography-mass spectrometry (LC-MS) technique was used to study fecal metabolomics to identify the changed metabolites. Identifying gut microbiota metabolites provides new ideas for further studies on the changes in gut microbiota in CIA mice and RA prevention and treatment.

## Materials and methods

### Feeding and grouping of experimental animals

In this study, we experiments were conducted on 32 four-week-old Specific-pathogen-free (SPF) DBA/1 mice. All the mice were male and weighed 14–16 g (experimental animal production license number: SCXK (Jing) 2014-0004; experimental animal use license number: SYXK (Liao) 2018-0008). The animals were provided by Huafukang Biotechnology Co., Ltd. and raised in the animal experiment center of China Medical University. The mice were kept in cages at 20–25 °C, with 40–60% relative humidity, in a room adjusted to a 12-h light/dark cycle. They were allowed free-access to standard food and water.

After one week of adaptive feeding, the mice were randomly divided into two groups of 16 according to their body weight. One group was given a normal diet, and the other group was fed on HFD. After five weeks of continuous feeding, eight mice were randomly selected from the two groups for CIA modeling. Finally, the mice were divided into four groups:

N Group: normal diet

C Group: normal diet + CIA

T Group: HFD

CT Group: HFD + CIA

All four groups were allowed free access to water, and each group continued to be fed with the above feed for 10 weeks.

### Reagents and materials

#### Feed composition

**Basic feed:** the energy supply ratio of fat, which is about 10%, was provided by the animal experiment center of China Medical University.

**High-fat feed (h10060):** the energy supply ratio of fat, which is about 60%, containing 26% protein, 26% carbohydrate and 35% fat, was provided by Huafukang Biotechnology Co., Ltd (Beijing, China).

#### CIA reagent preparation

To prepare the CIA reagent, chicken type II collagen (CII; Chondrex, Inc., Redmond, WA, USA) was dissolved in 0.02 mol/L^−1^ glacial acetic acid solution under sterile and dark conditions to prepare a solution with a final concentration of 2 mg/ml. The solution was ground, stirred evenly and placed in a 4 °C refrigerator overnight. The next morning, the chicken II collagen solution and an equivalent volume of complete Freund’s adjuvant (CFA; Chondrex, Redmond, WA, USA), both at a concentration of 4 mg/ml, were drawn repeatedly into a syringe over the course of one hour. This process was performed under consistent conditions on an ice bath. The sucked solutions were placed in a mortar and ground for about one hour to form a white emulsion. This white emulsion is the standard for complete emulsification of the CIA model reagent. This means that the white emulsion is dropped into the water and allowed to stand for five to ten minutes without spreading.

#### Preparation of weigert stain solution

The Weigert dye solution [Solarbio Science & Technology Co., Ltd, Beijing, China] used in this study was prepared by mixing equal amounts of Weigert A and Weigert B solutions.

### Animal treatment

The mice were immunized at the 5th week of feeding (10 weeks old). The first immunization was performed on day zero. The C group and the HFD mice subjected to CIA modeling were injected with the CIA model reagent emulsified by chicken type II collagen solution and equal volume CFA. The mice were injected at multiple points in the skin of the tail root at the dose of 0.15 ml/mouse reagent under sterile conditions. The second immunization was performed at 3 weeks after the first immunization (13 weeks of age) with the 0.1 ml/mouse of reagent. The procedure for subsequent immunization was consistent with the initial immunization protocol [71, 72]. Mice in both the N and T groups received injections of the same dose of normal saline using identical methods and timing.

One day after the secondary immunization, the limbs of mice were scored by two independent observers according to the Arthritis Index (AI) scoring method [73, 74]. Subsequently, the limbs were examined and recorded every three days. According to the five-point scale of 0–4, the sum of the joint score for each limb is the arthritis index. The AI score for each mouse is up to 16 points, and an AI score ≥ 1 indicates successful modeling.

### Assessment of arthritis

#### Enzyme-linked immunosorbent assay (ELISA)

Blood samples were collected from the orbital sinus of the mice, and were immediately transferred into ethylenediaminetetraacetic acid (EDTA)-coated tubes for analysis, then centrifuged at 3000 rpm for 10 min at 4 °C. Subsequently, 200 μL of serum was used to detect lipids [total cholesterol (TC), triglyceride (TG)] using the ELISA kits (Nanjing Jiancheng Bioengineering Institute, Nanjing, China, Catalog Nos. A111-1-1 and A110-1-1). Briefly, the samples were then incubated with the specific anti-TC and anti-TG antibodies provided in the ELISA kit, following the manufacturer’s instructions. After the incubation period, the wells were washed to remove unbound components. A horseradish peroxidase (HRP)-conjugated secondary antibody was added to each well, facilitating the detection of the bound serum lipids. The reaction was developed using a tetramethylbenzidine (TMB) substrate, which turns blue in the presence of HRP and changes to yellow upon the addition of the stop solution. The optical density of each well was measured at 450 nm using a microplate reader. This absorbance is directly proportional to the concentration of serum lipids in the samples, allowing for the quantification of TC and TG levels. The results were analysed using the standard curves generated from the known concentrations of the lipid standards provided in the kit.

#### Histopathological examination

Mice were sacrificed by decapitation and the hindlimb ankle joints were amputated. The surrounding skin and muscles were removed, and the intact ankle joint and surrounding synovial tissue were preserved. The joints were fixed in 4% paraformaldehyde, decalcified in 10% EDTA [Solarbio Science & Technology Co., Ltd, Beijing, China], and embedded in paraffin. Sections were then stained with hematoxylin-eosin (H&E) and Safranin O fast green staining. Hematoxylin stain [Zhuhai Besso Biotechnology Co., Ltd, Zhuhai, China.] Eosin Y Stain [Zhuhai Besso Biotechnology Co., Ltd, Zhuhai, China.] Modified Safranin O fast green FCF Cartilage Stain Kit [Solarbio Science & Technology Co., Ltd, Beijing, China]. The HE-stained sections were placed under a microscope. The histopathological alterations in the joints were evaluated by two observers who adopted a double-blind semi-quantitative scoring approach [75]. Moreover, to improve the reliability of our findings, we averaged the scores assigned independently by two researchers. The joint pathology score was graded on a scale of 0–4 (0: normal joint synovium and bone; 1: FLS proliferation and inflammatory cell infiltration; 2: pannus formation and cartilage erosion; 3: extensive cartilage and bone damage; 4: joint adhesions and disability). The cartilage erosion score ranged from 0 to 4 (0: no cartilage erosion; 1: minimal erosion less than 10% of the articular cartilage; 2: erosion up to 30% of the articular cartilage; 3: erosion up to 50% of the articular cartilage; 4: severe erosion over 50% of the articular cartilage) [76, 77].

#### Western blotting assay

The ankle joint was ground into powder and split with lysate for 1 h. After that, the concentration of bicinchoninic (BCA)protein assay Beyotime Biotechnology, Shanghai, China] was measured. Subsequently, the protein was boiled and packed separately. Then SDS-PAGE glue [Beyotime Biotechnology, Shanghai, China] was prepared, and an electrophoresis buffer was added. The protein was loaded at a volume of 20 μL per hole. The polyvinylidene fluoride (PVDF) membrane was cut, soaked in methanol for 10 s, and placed on the filter paper for wet transfer. After wet transfer, the PVDF membrane was blocked at room temperature for 30 min. The PVDF membrane was incubated with the corresponding protein antibodies: glyceraldehyde-3-phosphate dehydrogenase (GAPDH) (1:1000, Affinity, jiangsu, china, T0004), NOD-, LRR- and pyrin domain-containing protein 3 (NLRP3) (1:1000,Cell Signaling, Massachusetts, USA, 15101S), Interleukin 6 (IL-6) (1:1000, Affinity, jiangsu, china, DF6087), Interleukin 18 (IL-18)(1:1000), and interleukin-1beta (IL-1β) (1:1000, Cell Signaling, Massachusetts, USA, 12703S). This was followed by incubation with secondary antibody (1:20,000) for 2 h. The secondary antibodies used were Goat Anti-Rabbit IgG (H + L) and Goat Anti-Mouse IgG (H + L), both HRP-conjugated, with a dilution of 1:3000. These antibodies were obtained from Affinity (Jiangsu, China). The protein blots were detected using enhanced chemiluminescence (ECL). Protein expression was quantification by Image J software (1.48, National Institutes of Health, America).

### 16S rRNA amplicon sequencing of gut microbiota

#### Fecal specimens and tissue specimens are retained

At the end of the trial (on the 35th day after initial immunization), at least two fecal samples were collected from the colon and stored at −80 °C for 16S rRNA amplicon sequencing and fecal metabolic profiling.

#### 16S rRNA amplicon sequencing experimental method

##### DNA extraction and amplification

Total genomic DNA was extracted using MagPure Soil DNA LQ Kit (Magan) following the manufacturer’s instructions. The bacterial 16S rRNA gene was amplified using PCR with barcoded primers and Takara Ex Taq (Takara). The bacterial diversity analysis was performed using the V3-V4 variable regions of 16S rRNA genes. These regions were amplified using universal primers 343F (5’-TACGGRAGGCAGCAG-3’) and 798R (5-AGGGTATCTAATCCT-3’) for V3–V4 regions [78].

##### Library construction and sequencing

The Amplicon quality was visualized using agarose gel electrophoresis. The PCR products were purified using AMPure XP beads (Agencourt) and amplified through another round of PCR. The Amplicon quality was visualized using agarose gel electrophoresis. After purification with the AMPure XP beads, the final amplicon was quantified using Qubit dsDNA Assay Kit (Thermo Fisher Scientific, USA). The Illumina NovaSeq 6000 was sequenced with an end-reading of 250 bp (Illumina Inc., San Diego, CA; OE Biotech Company, Shanghai, China).

##### 16S rRNA amplicon sequencing analysis process

The original sequencing data was in FASTQ format. The paired-end readings were then preprocessed using Trimmomatic [79] software, a flexible trimming tool for Illumina NGS data. After trimming, pairs of end readings were assembled using FLASH software [80]. In addition, the QIIME software (version 1.8.0) was used to detect denoising and exclude readings with chimeras [81].

Vsearch [82] software was used to remove primer sequences and cluster them to generate operational taxonomic units (OTUs). All representative readings were annotated according to Silva database version 138 using the RDP classifier [83] with a confidence threshold of 70%.

The QIIME software was used for α and β diversity analysis. Microbial diversity in samples was estimated using α diversity, including the Chao1 index [84] and Shannon index [85]. Principal coordinate analysis (PCoA) was used to estimate β diversity. The ANOVA/Kruskal-Wallis statistical test analyzed the significant differences between the groups. The linear discriminant analysis Effect size (LEfSe) method was used to compare the taxonomic abundance spectra among various species.

### Fecal metabolic profiling

#### Sample preparation

The samples were first thawed and added to 1.5 mL Eppendorf tubes, with 20 μL L-2-chlorophenyl alanine (0.06 mg/mL) dissolved in methanol as an internal standard. The solution was then rotated for 10 s. Subsequently, an icy mixture of 600 μL methanol and acetonitrile (2/1, vol/vol) was added and rotated for 1 minute. The whole sample was extracted using ultrasound in an ice water bath for 10 min and stored at −20 °C for 30 min. The sample was centrifuged at 13,000 rpm for 10 min at 4 °C (13,000 rpm). The supernatant was dried. After drying, 300 μL of the supernatant was mixed with 400 μL methanol and water (1/4, vol/vol) and stored at −20 °C for two hours. The solution was centrifuged at 13,000 rpm for 10 min at 4 °C, and 150 μL of the supernatant was carefully collected. It was then filtered using a 0.22 μm microfilter and transferred into an LC bottle for storage at −80 °C until LC-MS analysis.

#### LC-MS analysis

Metabolomics data analysis was performed at Shanghai Deer Sing Biotechnology Co., Ltd. (Shanghai, China). ACQUITY UPLC I-Class plus (Waters Corporation, Milford, USA) equipped with Q-Exactive mass spectrometer and a heated electrospray ionization (ESI) source (Thermo Fisher Scientific, Waltham, MA, USA) was used for the analysis of ESI positive and ESI negative ion modes. On the other hand, the ACQUITY UPLC HSS T3 column (1.8 μm, 2.1 × 100 mm) was used for positive and negative mode analysis.

#### Data preprocessing and statistical analysis

The Human Metabolome Database (HMDB), Lipidmaps (V2.3), Metlin and self-established databases were used to identify compounds.

A matrix was introduced into R for principal component analysis (PCA) to observe the overall distribution among samples and the stability of the analysis process. Partial least squares discriminant (PLS-DA) was used to distinguish different metabolites in the groups. Two-tailed Student’s T-test was used to verify differences. VIP values greater than 1.0 and *p*-values less than 0.05 were used as criteria to select the metabolites.

#### Pearson correlation algorithm

The Pearson correlation algorithm was used to calculate the correlation between microbial abundance and metabolite reaction intensity.

### Statistical analysis

Data are presented as means ± standard deviation (SD). All statistical analyses were performed using SPSS 22.0 (IBM Corp, Armonk, New York, US) and GraphPad Prism 8 software (GraphPad Software, Inc., La Jolla, CA, USA). The Shapiro-Wilk test was used to assess data normality, whereas homogeneity of variance was tested using the Levene method. Normally distributed data exhibiting homogeneous variance were analyzed with Student’s t-tests or one-way ANOVA with Tukey’s test. All other data were analyzed with non-parametric Kruskal-Wallis or Mann-Whitney tests. *p* < 0.05 was the significance threshold.

## Results

### HFD exacerbates arthritis index (AI) scores in CIA mice

**Fig. 1:**
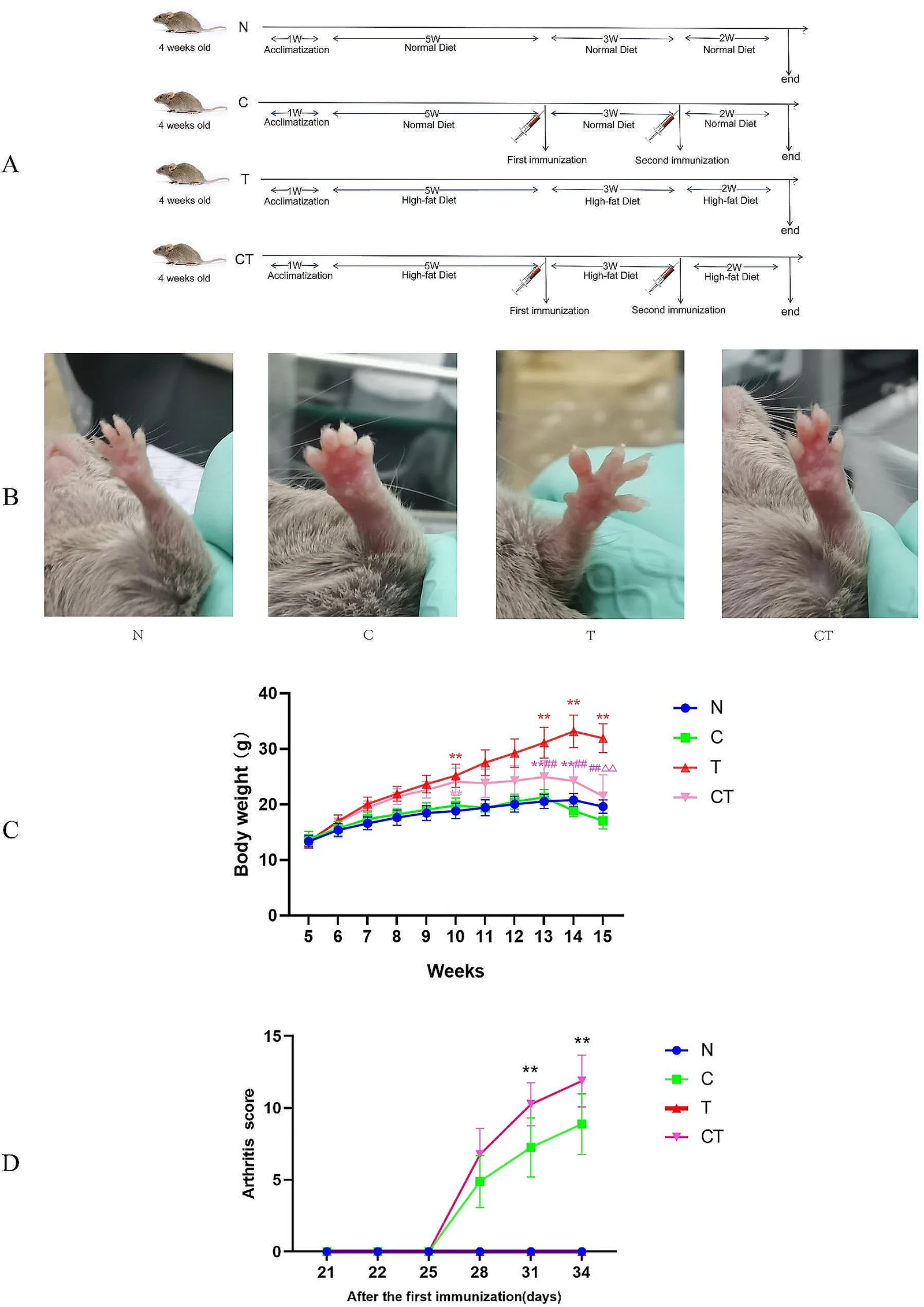
Effect of HFD on arthritis response in CIA mice. (**A**) Procedure of the experiment. (**B**) Representative imaging for ankle joints within individual groups. (**C**) Body weight. (**D**) Arthritic score. n = 8 per group. *P < 0.05, **P < 0.01 vs N group; ^#^*P* < 0.05, ^##^*P* < 0.01 vs T group; ^Δ^*P* < 0.05, ^ΔΔ^*P* < 0.01 vs C group.

The CIA model of mice was established by administration of CII into CFA to verify the effect of a HFD on arthritis. The first immunization was done when the mice were 10 weeks old. This was recorded as day 0. The second immunization was on day 21 (13 weeks old). Ten days after the second immunization (day 31), the paws of the mice were red and swollen. The swelling first appeared on the primary side and then extended to the secondary side, tail, and ear. The experimental procedure is illustrated in Fig. 1A. Macroscopic observation showed that the peak of swelling was on day 34 (Fig. 1B).

The mice were weighed once every week after the 5th week to determine the effect of a HFD on arthritis in CIA mice. Figure 1C shows that there was no difference in the body weight of mice in each group at the start of the experiment. After five weeks of feeding, the growth rate of mice in the HFD was faster than that of the mice in the normal diet group. After 11 weeks, the body weight of arthritis model mice showed a decreasing trend. The weight gain of mice in the CT group was significantly lower compared to the T group (*P* < 0.01). Moreover, the weight of mice in both the CT and T groups was higher than in the N group (*P* < 0.01). Between weeks 13 and 14 (21 days after the initial immunization), the body weight of mice in the C and CT groups declined, while that of the T group increased significantly. By weeks 14–15, the weight of mice in all groups decreased.

Figures 1D show mice in N and T groups had no arthritis, and their AI was always 0. The results also show that the mice in C and CT groups had joint swelling 28 days after the first immunization. The AI score of mice in C and CT groups had a continuous growth trend. From the 31st day, the AI score of mice in the CT group was significantly higher than that in the C group (*P* < 0.05).

### HFD exacerbates histopathological deterioration in CIA mice

**Fig. 2:**
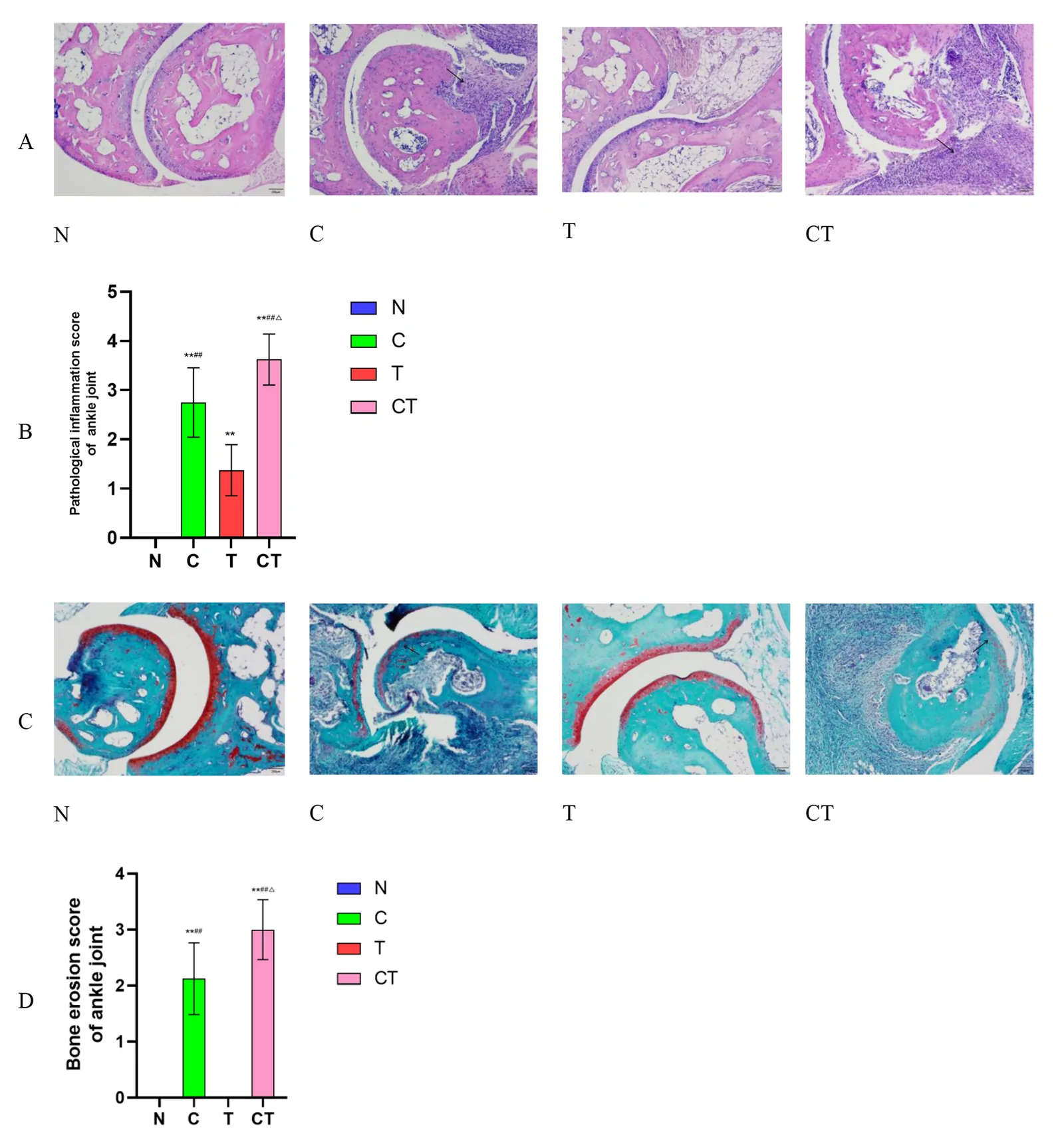
The effect of HFD on arthritis response in CIA mice. (**A**) H&E staining images of joint tissue (scale bar, 250 μm) (**B**) Mouse ankle synovitis score (**C**) Safranin O-Fast Green Staining (scale bar, 250 μm) (**D**) Ankle bone erosion score in mice. The position indicated by the arrow is the location of inflammatory cell infiltration. n = 8 per group. *P < 0.05, **P < 0.01 vs N group; ^#^*P* < 0.05, ^##^*P* < 0.01 vs T group; ^Δ^*P* < 0.05, ^ΔΔ^*P* < 0.01 vs C group

Figure 2A displays the HE staining of the ankle tissues. In the N group, joint tissue sections revealed clear, smooth articular surfaces with intact and uniform joint spaces, and the cartilage on the outer side of the joints featured neatly arranged single or double layers of synovial cells. Compared to the N group, the T group showed minimal inflammatory cell infiltration, with no evidence of synovial proliferation, pannus formation, bone erosion, or cartilage destruction observed. In contrast, both the C group and the CT group exhibited significant synovial cell proliferation. The synovial layers were increased and disorganized, accompanied by fibrous tissue proliferation, extensive inflammatory cell infiltration, pannus formation, and severe cartilage damage.

The synovitis score of ankle tissue sections in each group is shown in Fig. 2B. Compared to the N group, the synovitis score of the C, T, CT group was significantly higher (*P* < 0.01). Further analysis revealed that the synovitis score of the CT, C group was significantly higher relative to that of the T group (*P* < 0.01). Lastly, the synovitis score of the CT group was significantly higher than that of the C group (*P* < 0.05).

Figure 2C shows the pathological picture of ankle cartilage tissue of mice in each group after safranin o-solid green staining, where cartilage appears red and bone and muscle tissues appear green. The N group shows complete cartilage with uniform thickness and clear contours, while the T group has uniformly thick structures with slightly lighter staining but clear boundaries. Conversely, in the C and CT groups, there is a significant reduction in red staining intensity of the cartilage, indicating severe degradation and compromised tissue integrity, highlighting the destructive impact of the pathological conditions on cartilage.

The ankle cartilage erosion score of the N and T groups of mice was 0. Compared to the N and T group, the ankle cartilage erosion in C group and CT groups was significantly higher (*P* < 0.01). In addition, the evaluation of ankle cartilage erosion in the CT group was significantly higher than that in the C group (*P* < 0.05) (Fig. 2D).

### HFD exacerbates the expression of inflammatory inflammatory cytokines in CIA mice

**Fig. 3:**
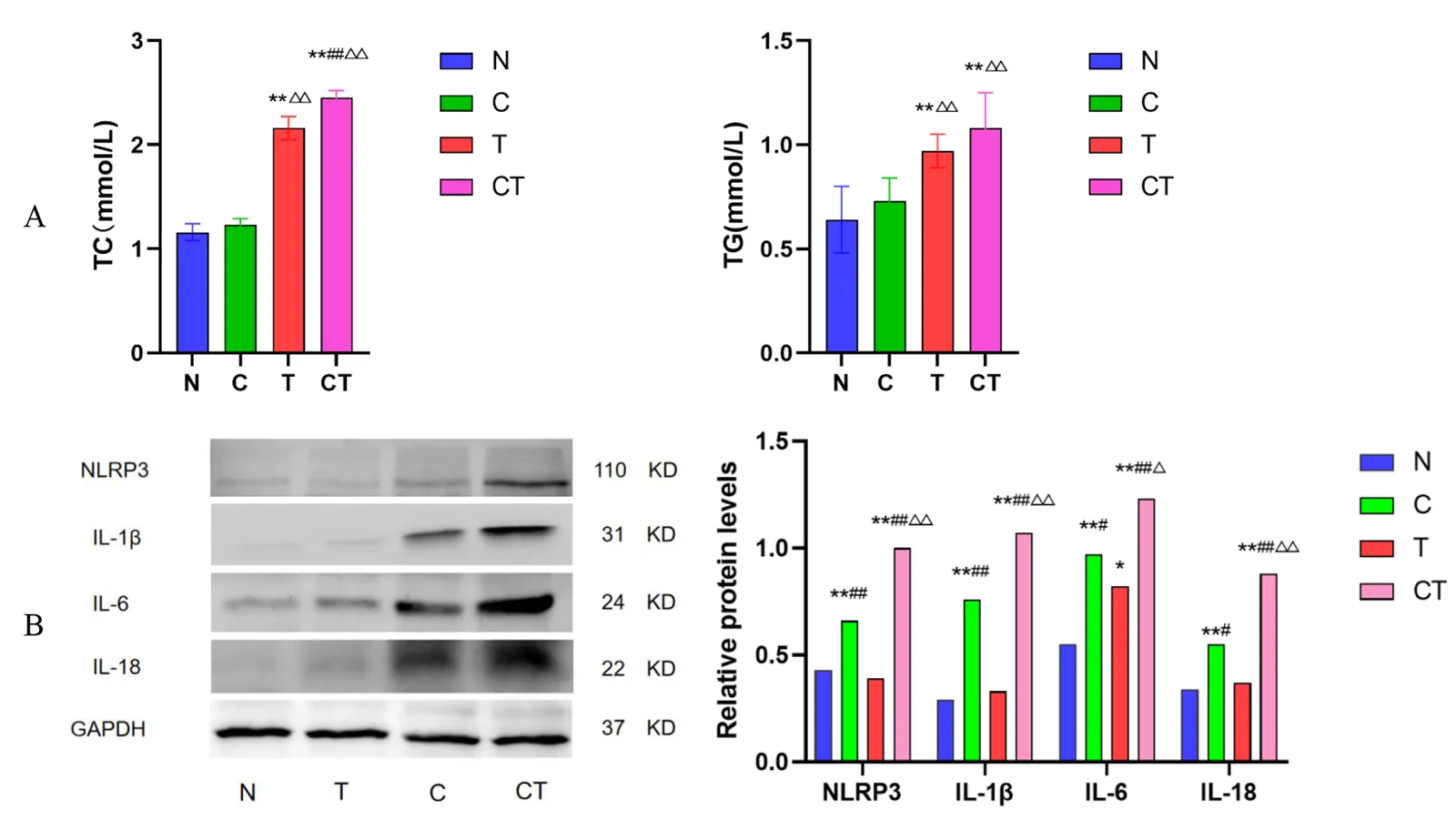
Effects of HFD on TC,TG and inflammatory cytokine levels in CIA mice. (**A**) Serum level of TC and TG (n = 8). (**B**) The protein expression of NLRP3, IL-β, IL-6, IL-18 (n = 3). Data presented as the means ± SD. Significant differences were indicated as *P < 0.05, **P < 0.01 vs N group; ^#^*P* < 0.05, ^##^*P* < 0.01 vs T group; ^Δ^*P* < 0.05, ^ΔΔ^*P* < 0.01 vs C group. *TC* total cholesterol, *TG* triglyceride

Figure 3A shows that serum TG and TC levels were lower in the N group than in the T group (*P* < 0.01). In addition, the levels of TG and TC in the C group were significantly lower than in the CT group (*P* < 0.01). The levels of serum TC in the CT group were higher than in the C and T groups (*P* < 0.01).

There was no significant difference in the relative expression of NLRP3, IL-1β, and IL-18 in the ankle joint tissue of the T group and the N group, as shown in Fig. 3B. The relative expression of IL-6 protein in the ankle joint was higher in the T group mice than in the N group(*P* < 0.05). Moreover, the relative expression of NLRP3, IL-1β, IL-6, and IL-18 proteins in the ankle joint tissue of the C and CT groups was significantly higher than in the N group (*P* < 0.01). Unlike the C group, the relative expressions of NLRP3 (*P* < 0.01), IL-1β (*P* < 0.01), IL-6 (*P* < 0.05), and IL-18 (*P* < 0.01) proteins in the ankle joint tissue were higher in the CT group. Compared with T group, the expression of NLRP3 (*P* < 0.01), IL-1β (*P* < 0.01), IL-6 (*P* < 0.05), and IL-18 (*P* < 0.05) proteins in group C was significantly increased, and the expression of NLRP3 (*P* < 0.01), IL-1β (*P* < 0.01), IL-6 (*P* < 0.01), and IL-18 (*P* < 0.01) proteins in group CT was significantly increased.

The results showed that FHD aggravated the severity of arthritis in mice. However, the mechanism of HFD needs to be explored further.

### Effects of HFD on gut microbiota diversity of CIA mice

**Fig. 4:**
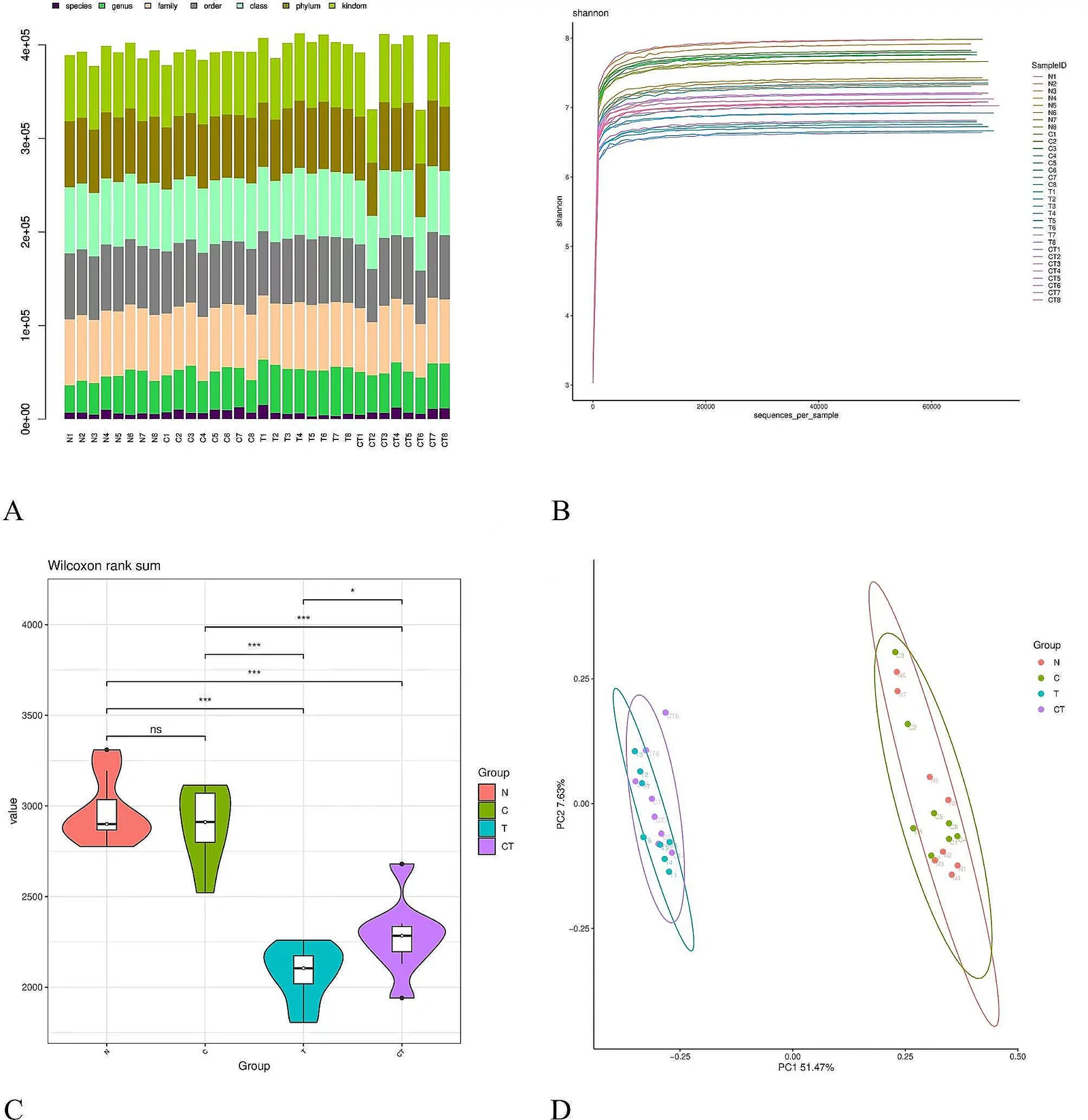
Diversity analysis of the gut microbiota among different groups. (**A**) The OTU level bar plot reflects the average number of tags in OTUs. A column represents a sample, and the vertical axis is the average number of tags in OTUs under different classification levels. (**B**) Shannon rarefaction curve in individual groups. (**C**) Alpha diversity index (Chao estimator) analysis. Student’s t-test was employed to determine the discrepancies. (**D**) PCoA for unweighted UniFrac distance metrics among four groups in gut microbiota populations. n = 8 per group. Significant differences were indicated as *P < 0.05, **P < 0.01, ***P < 0.001

The composition spectrum of gut microbiota was analyzed using 16S rRNA gene sequencing. A total of 1,437,216 clean readings from 32 samples were sequenced to determine the effect of HFD on the gut microbiota composition of CIA mice. The OTU level bar plot reflects the average number of tags in OTUs under different classification levels (Fig. 4A). The curve is consistent with the Shannon rarefaction map, whose curve tends to be flat. The sequencing depth covered all species of the inverted sample, indicating that the sequencing range can determine the biodiversity of the whole sample (Fig. 4B).

The Chao diversity index indicates the distribution of diversity within various groups. This index can help to explore whether there are significant variations in diversity among the groups. In this study, diversity analyses revealed significant differences among all the four groups. Particularly, the diversity curve for T group was lowest. The Chao index of α diversity showed that HFD reduced the heterogeneity of the gut microbiota in the T and CT groups than in the N and C groups (Fig. 4C).

The principal coordinate analysis (PCoA) was performed to compare bacterial community patterns among the groups. The two principal coordinates with the biggest difference between the samples were the abscissa PC1 and ordinate PC2. The PCoA scores of OTU horizontal sequences (similarity > 59%) showed significant differences in gut microbiota diversity between the four groups after HFD treatment. PCoA using UniFrac distance identified differences in microbiome composition between the T and CT groups contrasted with the N and C groups (R = 0.7216, *P* = 0.0010). Moreover, significant differences were recorded within the gut microbiota of the CT and T groups. Specifically, the PCoA revealed a more pronounced distribution of coordinate distribution within the CT group compared to the T group (R = 0.6461, *P* = 0.0010) (Fig. 4D). These findings suggest that HFD alters the gut microbiota structure of CIA mice.

### Effects of HFD on the changes of microbiota and genus levels in CIA mice

Community structure is mainly used to present the overall microbial composition and display the information of dominant flora. The histogram is drawn according to the relative abundance percentage of species from the statistical analysis.

**Fig. 5:**
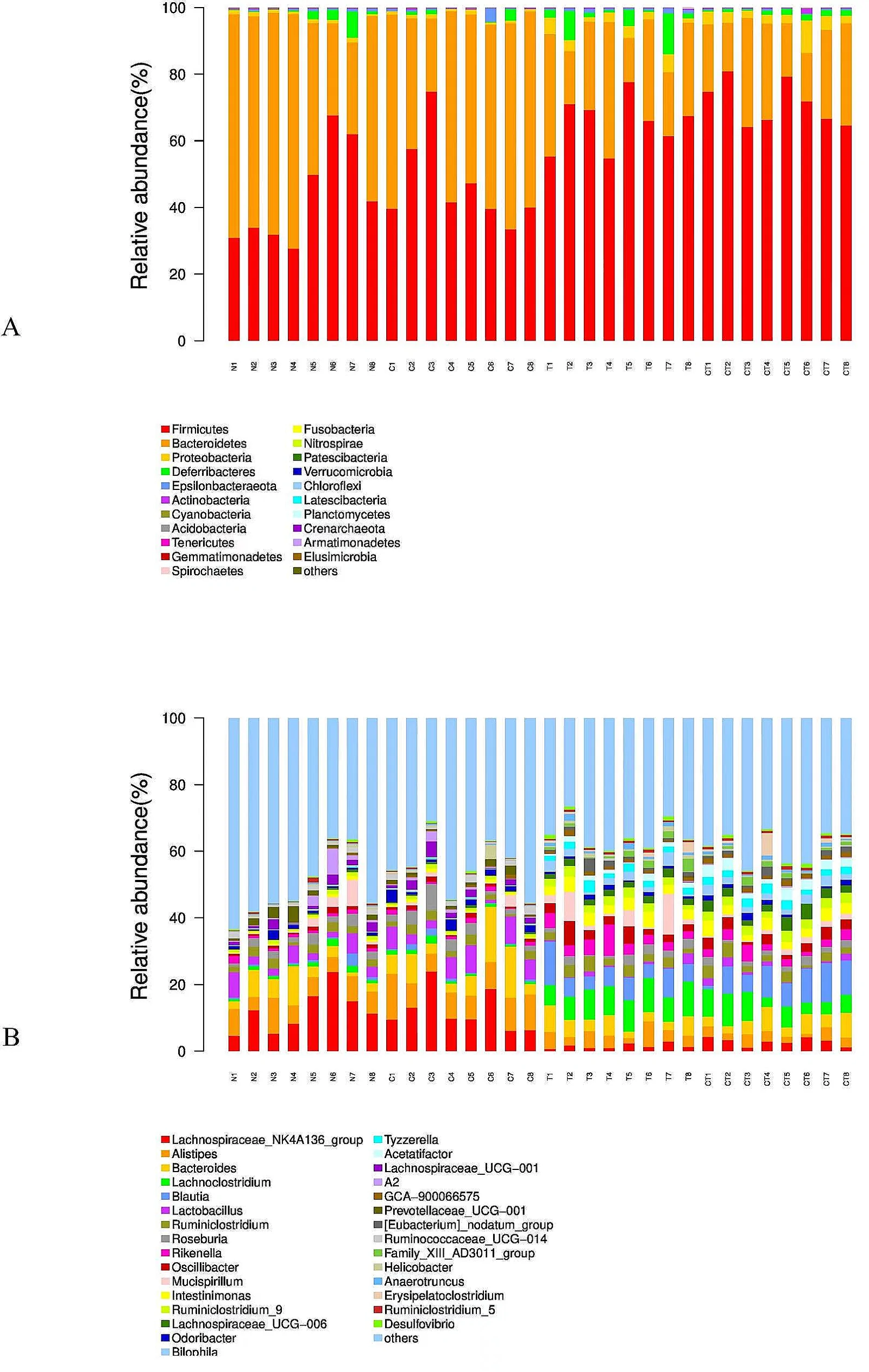
Related phylum- and genus-level bacterial abundance. (**A**) Stacked bar plot of the Top 30 at the phylum level. (**B**) Stacked bar plot of the Top 30 at the genus level. n = 8 per group

The results demonstrated that *Firmicutes, Bacteroidetes, Proteobacteria, Deferribacteria, Epsilonbacteraeota*, and *Actinobacteria* were relatively enriched at the phylum level. Group T compared to group N, group CT compared to group C, high-fat factor significantly increased *Firmicutes* and *Proteobacteria* and decreased *Bacteroidetes*. Group CT compared to group T, group C compared to group N, the number of *Deferribacteres* was significantly decreased due to the CIA compound model factor (Fig. 5A).

*Lachnospiraceae* NK4A136 group, *Alistipes, Bacteroides, Lachnoclostridium, Blautia, Lactobacillus, Ruminiclostridium, Roseburia*, and *Desulfovibrio* were relatively enriched at the genus level. Group T compared to group N, group CT compared to group C, the high-fat factor significantly increased *Lachnoclostridium* and *Blautia* and decreased *Lachnospiraceae* NK4A136 group, *Alistipes, Bacteroides, Lactobacillus*. Moreover, group T compared to group CT, the CIA model factors significantly increased *Lactobacillus* and decreased *Lachnoclostridium* and *Lachnospiraceae* UCG-006 (Fig. 5B).

### Effect of HFD on core gut microbiota in CIA mice

Through Linear discriminant analysis Effect Size(LEfSe) analysis, we explored the species composition within diverse biological community groups. The LEfSe approach presents each group with a different color, and uses colored dots to indicate species with increased abundance and significantly differences across the groups. Each node in the visualization signifies taxonomic levels, ranging from phylum to family and genus, arranged progressively from the innermost to the outermost layers. We utilized the LEfSe method to identify metagenomic biomarkers through taxonomic comparison in order to ascertain the dominant bacterial taxa among various taxa. LEfSe is a novel approach for determining the most prevalent bacterial taxa. Further, 41 bacterial taxa were identified here with statistically significant and biologically consistent differences.

**Fig. 6:**
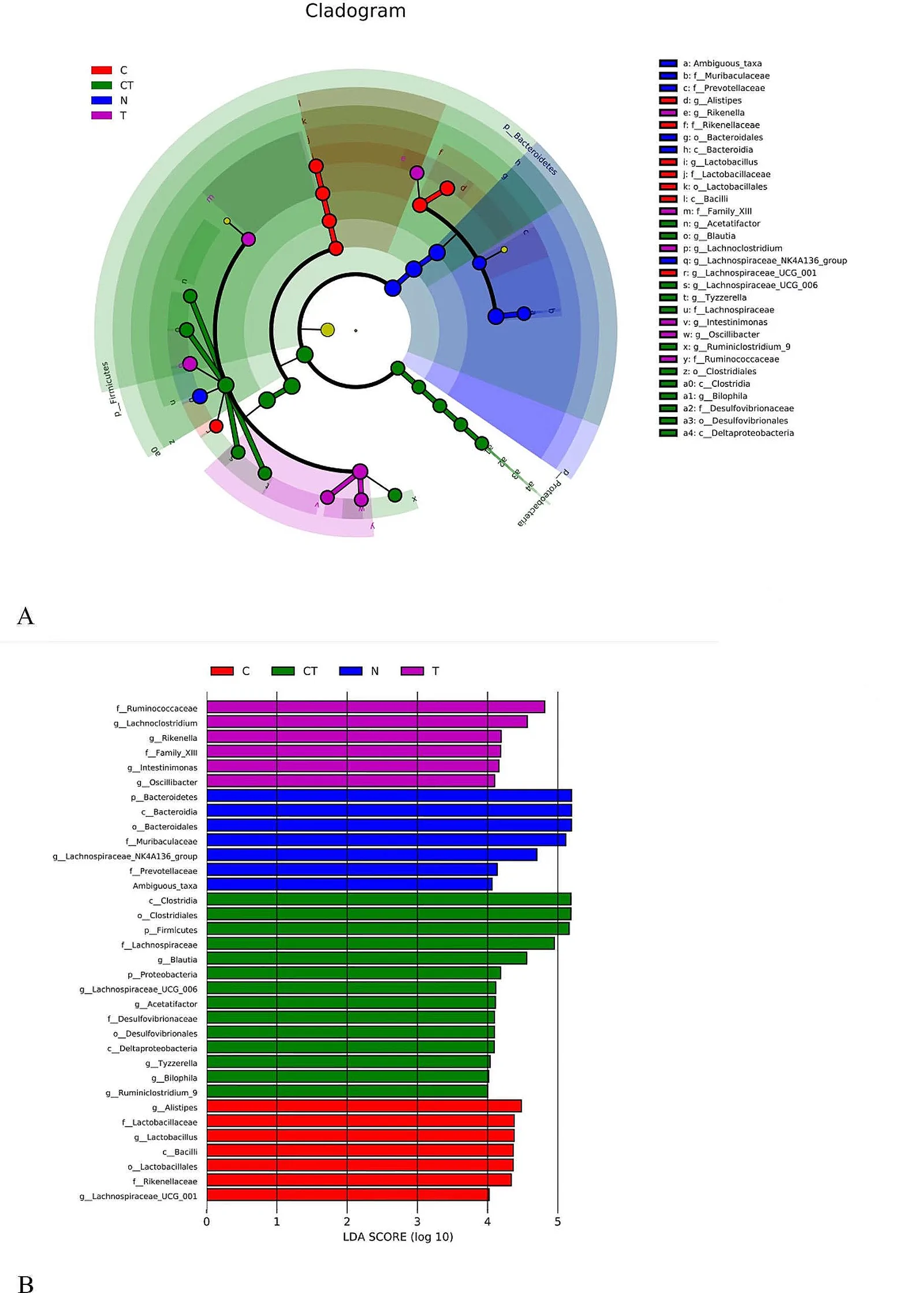
LEfSe: Species difference discriminant analysis. (**A**) By analyzing the generated taxonomic branch map, significant changes in the gut microbiota of each group were shown (score > 4). (**B**) Linear discriminant analysis (LDA) score (log10) of taxa (score > 4). n = 8 per group

The results showed that Ambiguous_taxa, f_Prevotellaceae, g_Lachnospiraceae NK4A136,f_Muribaculaceae, o_Bacteroidales, c_Bacteroidia, p_Bacteroidetes were abundant in the N group. The g_Lachnospiraceae UCG 001, o_Lactobacillales, c_Bacilli, f_Lactobacillaceae, g_Lactobacillus, f_Rikenellaceae and g_Alistipes were abundant in the C group. On the other hand, g_Oscillibacter, g_Intestinimonas, f_Family_XIII, g_Rikenella, g_Lachnoclostridium, and f_Ruminococcaceae were abundant in the T group. The g_Ruminiclostridium_9, g_Bilophila, g_Tyzzerella, c_Deltaproteobacteria, o_Desulfovibrionales, f_Desulfovibrionaceae, g_Lachnospiraceae UCG 006, g_Acetatifactor, p_Proteobacteria, g_Blautia, f_Lachnospiraceae, p_Firmicutes, o_Clostridiales, and c_Clostridia were abundant in the CT group (Fig. 6A, B).

**Fig. 7:**
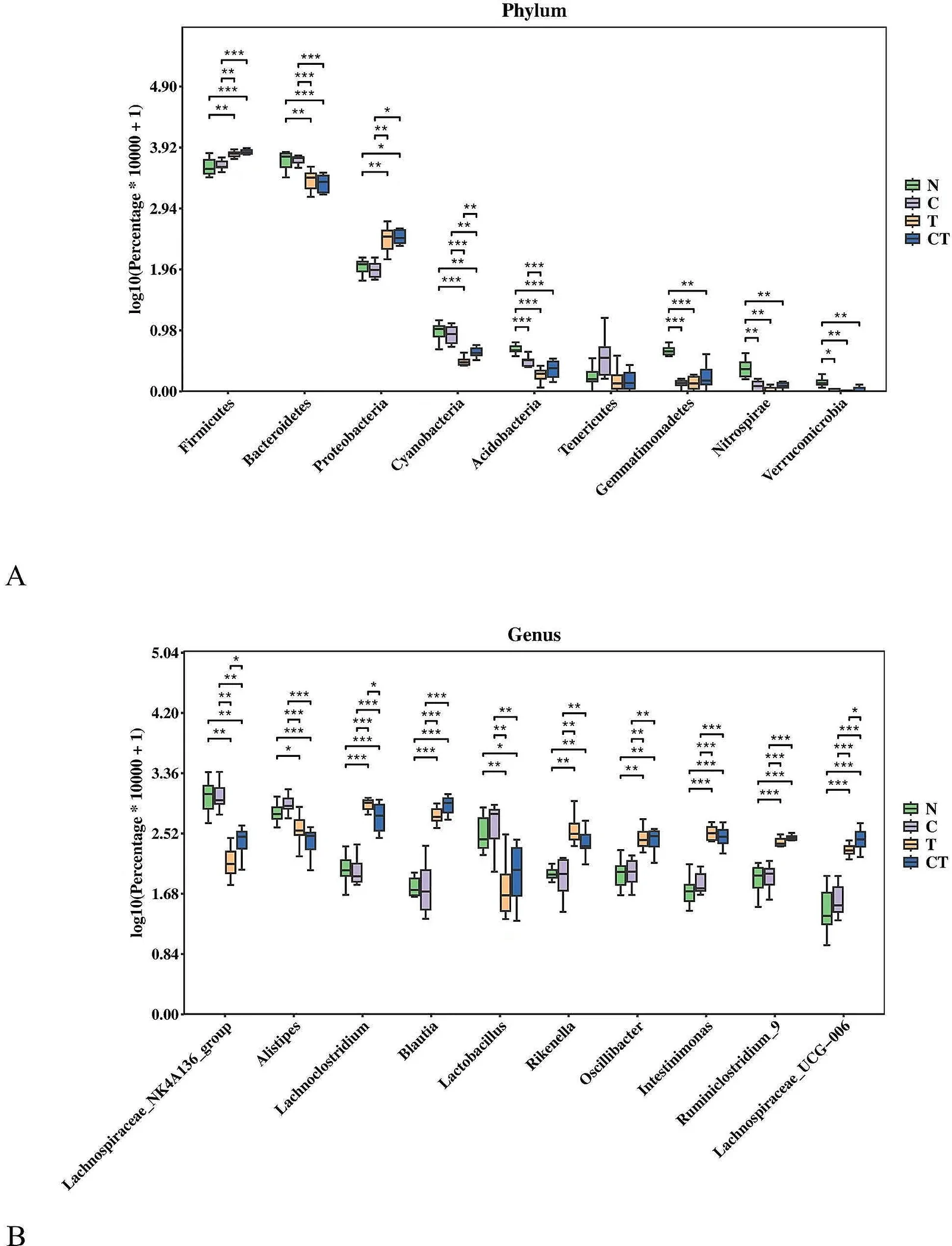
Boxplot of different species. (**A**) Boxplot map of 10 primary genera across all treatment classes. (**B**) Boxplot map nine primary phyla across all treatment classes. n = 8 per group. Significant differences were indicated as *P < 0.05, **P < 0.01, ***P < 0.001

To compare the dominant species across different groups, we selected the boxplot analysis of the top 10 species abundance in terms of relative abundance. In total, 10 phylum and 9 genera showed significant differences among the four groups. The changes in these box drawings are shown in Fig. 7A, B.

The proportion of microbial abundance of *Firmicutes* and *Proteobacteria* was higher in the CT than in all the other groups. In contrast, *Bacteroidetes* and *Verrucomicrobia* were lower in the CT group than in all the other groups at the phylum level (*P* < 0.05).

Compared with the other groups, the CT group had the highest proportion of microbial abundance of *Blautia, Oscillibacter, Ruminiclostridium-9, and Lachnospiraceae UCG 006* at the level of genus. Further analysis showed that the proportion of *Alistipes* was lower in the CT group than in all the other groups (*P* < 0.05).

This study suggested these changes in bacterial abundance may be the reason for the aggravated inflammation in the CT group.

### Effect of HFD on fecal metabolic profile of CIA mice

**Fig. 8:**
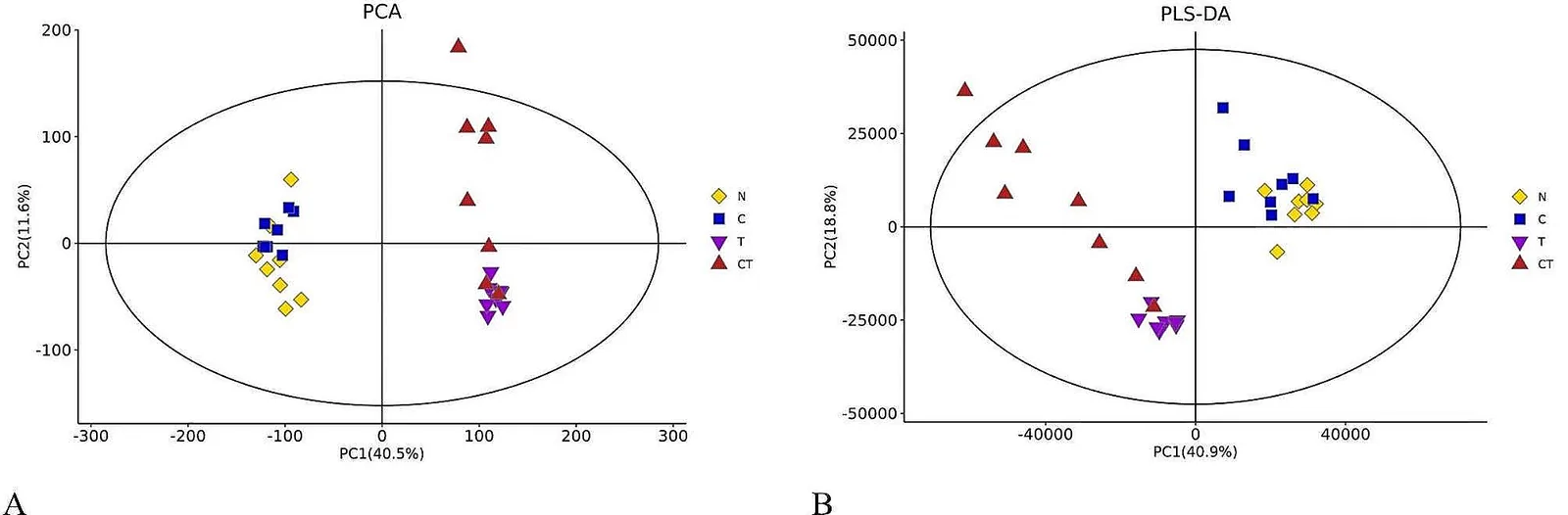
Overall distribution characteristics and stability analysis for various samples. (**A**) PCA score plots comparing the fecal metabolites in each group. (**B**) PLS-DA score plots comparing the fecal metabolites in each group. n = 8 per group. *P* values were evaluated using CV-ANOVA

To determine the effect of HFD on fecal metabolic profile of CIA mice, we plotted the intensity of the most significant ion in the mass spectrogram and displayed it as a chromatogram. Fecal metabolic base peak intensity curves of mice in each group after HFD feeding were generated using LC-MS as shown in Fig. S1 (Appendix). Notably, the base peak diagram shows the complete data of each group. PCA can provide important information about the initial state of the reaction data, allowing researchers to holistic understand the overall data status. To address the limitations of PCA, we used the partial least squares discriminant analysis (PLS-DA) which incorporated grouping variables based on PCA results. The projected scores of each sample on the plane formed by the first and second principal components were utilized as spatial coordinates, visually representing similarities or differences among samples. Widely distributed coordinate points indicated significant differences between samples, with the ellipse denoting a 95% confidence interval. Statistical analysis was performed on data from both positive and negative ions. Both PCA and PLS-DA models revealed significant differences in metabolic profiles between the N and C groups, as well as between the T and CT groups. These results demonstrated that HFD induced significant biochemical changes (Fig. 8A, B).

### Further screening of fecal differential metabolites of CIA mice by HFD

To further explore the effect of a HFD on fecal differential metabolites of CIA mice, the screening criteria for differential metabolites were: VIP value > 1, *P*-value of T-test < 0.05. The software UNIFI 1.8.1. was used to collect the original data of the biomarkers.

**Fig. 9:**
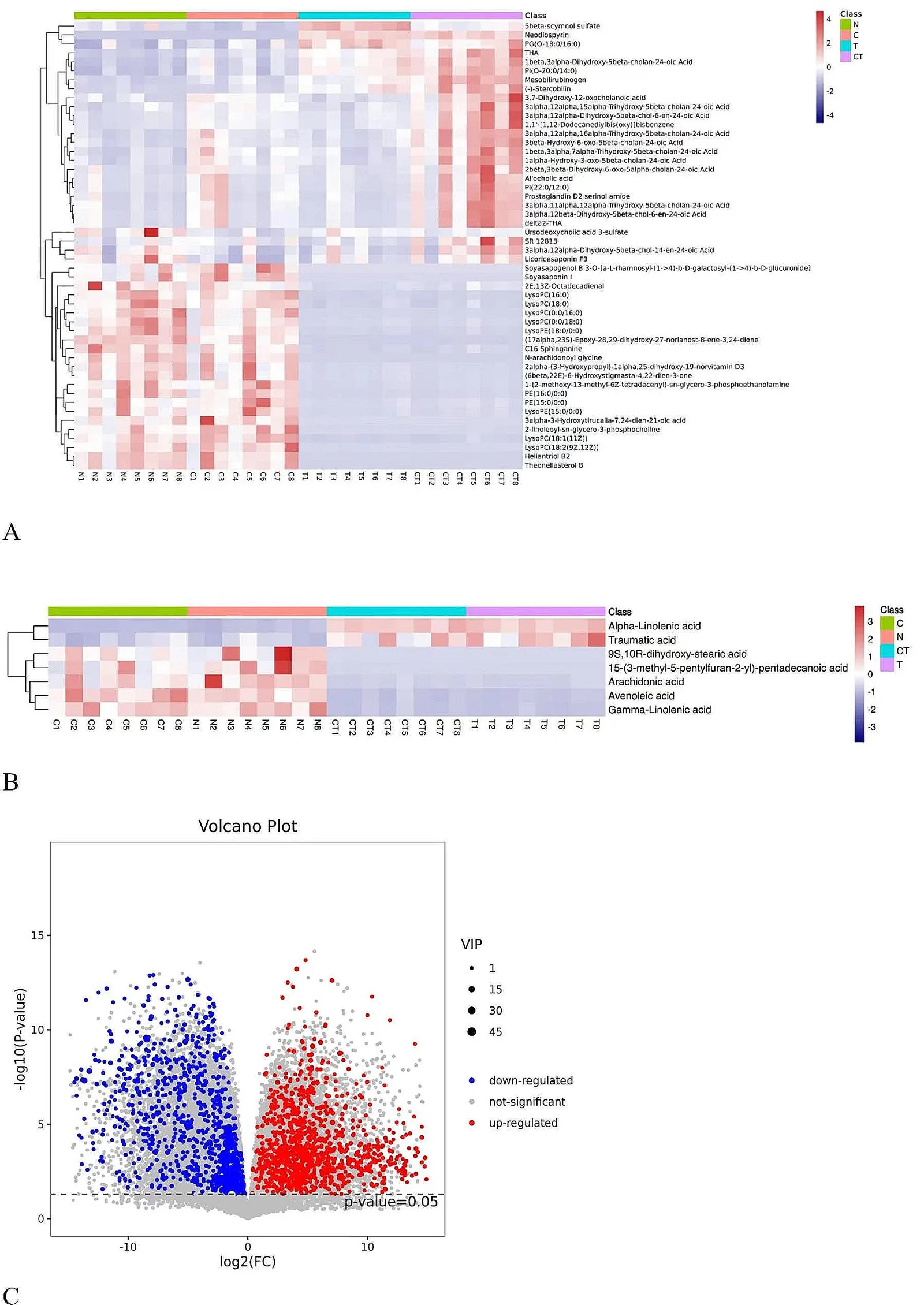
Differentially expressed metabolites in fecal samples. (**A**) Heatmap of the top 50 differentially expressed metabolites in each group (horizontal clustering). (**B**) Differentially expressed long-chain fatty acids among the four groups. (**C**) Volcano plot of differentially expressed metabolites in groups C and CT. n = 8 per group. Abscissa: metabolite difference multiple takes two as the bottom logarithm, Ordinate: significance takes 10 as the bottom negative logarithm. Red dots represent significantly up-regulated differential metabolites, blue dots represent down-regulated metabolites, gray dots represent insignificant differences in metabolites

A heat map was utilized to represent the top 50 differential metabolites across the four groups. In the heat map, colors ranging from blue to red denote varying levels of metabolite expression abundance, with darker reds indicating higher expression levels. In Fig. 9A, the marker section highlights the metabolites detected in the CT group, with oleic acid being the predominant metabolite. A stratified analysis of various categories of the obtained metabolites showed that levels of long-chain fatty acids, including Traumatic acid, Gamma-Linolenic acid, Oleic acid, Arachidonic Acid, Stearic acid, Alpha-Linolenic acid, and Pentadecanoic acid were significantly altered in T and CT group (*P* < 0.001). Specifically, high-fat factors significantly increased levels of Traumatic acid and Alpha-Linolenic acid in T and CT groups compared with N and C groups, as shown in Fig. 9B.

Volcanic maps are often used to visualize the p-value and fold change value, and thus suitable for the screening of differential metabolites. To further explore the effect of a HFD on metabolites in CIA mice, VIP and P screening volcano diagrams were constructed to reveal differences in metabolites between the C and CT groups in Fig. 9C.

### Screening of differentially expressed metabolic pathways in CIA mice after HFD

Metabolite enrichment analysis was conducted utilizing the KEGG database, with a significance threshold set at *p* ≤ 0.05. A lower *p*-value indicated significant enrichment. The results are shown by enrichment and bubble diagrams. In the enrichment map, the x-axis denotes the name of the metabolite pathway, while the y-axis represents the negative logarithmic value with a base of 10. The red line indicates that the *p* value is 0.01, and the blue line indicates that the *p* value is 0.05. Bubble plot: Enrichment factor is presented on the horizontal axis while the name of the metabolic pathway is presented on the vertical axis. The greater the enrichment factor, the greater the enrichment degree.

**Fig. 10:**
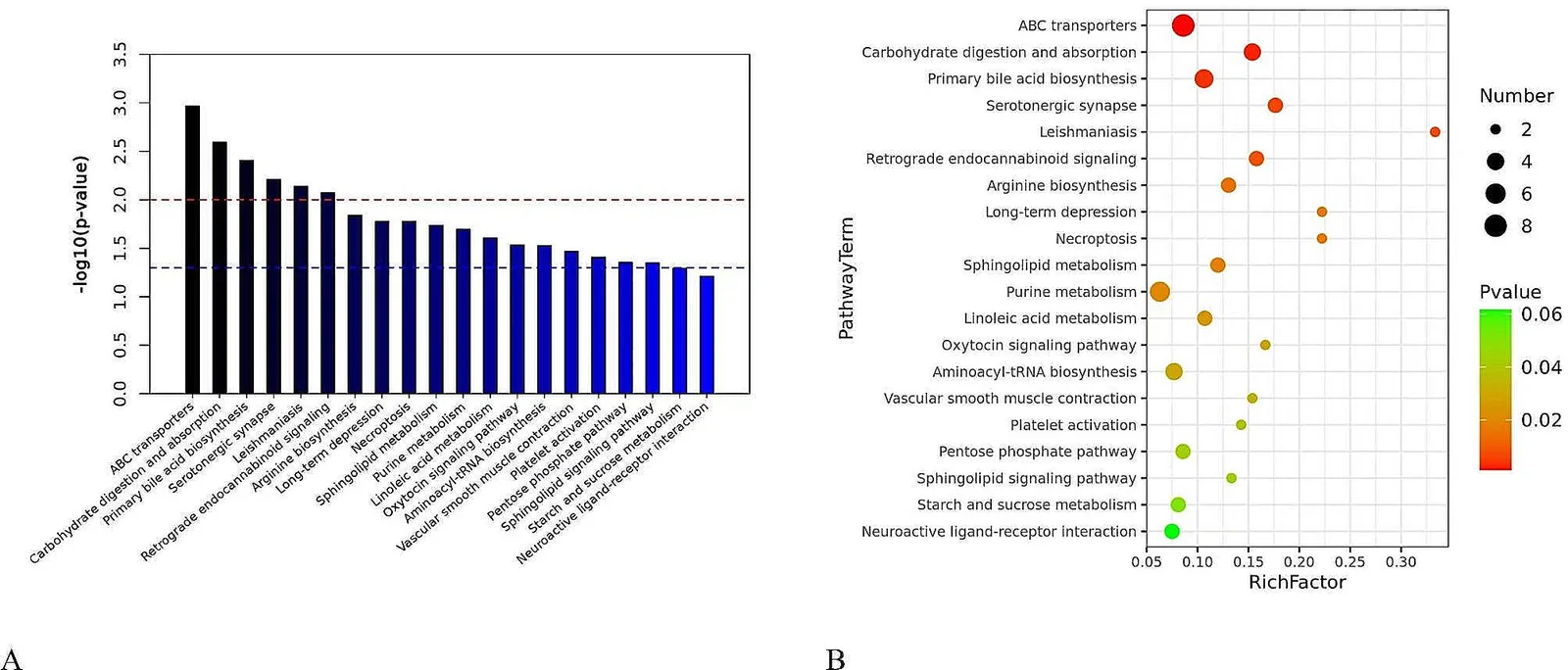
Significantly differentially expressed pathways in fecal samples (n = 8). (**A**) Enrichment diagram of differentially expressed metabolic pathways (*P* ≤ 0.05). (**B**) Bubble diagram of differentially expressed metabolic pathways (*P* ≤ 0.05)

Comparisons among the four groups revealed that 9 pathways were significantly differentially expressed (*P* ≤ 0.05), including, Primary bile acid biosynthesis (*P* = 0.003919), Arginine biosynthesis (*P* = 0.014541), Sphingolipid metabolism (*P* = 0.018281), Purine metabolism (*P* = 0.020081), Linoleic acid metabolism (*P* = 0.024802), Oxytocin signaling pathway (*P* = 0.029226), Aminoacyl-tRNA biosynthesis (*P* = 0.029600), Pentose phosphate pathway (*P* = 0.085714), Sphingolipid signaling pathway (*P* = 0.044499) (Fig. 10A, B).

### KEGG map

The KEGG map illustrates various enriched key metabolic pathways, such as primary bile acid biosynthesis, purine metabolism, linoleic acid metabolism, arginine biosynthesis, and pentose phosphate pathway. Differentially expressed metabolites are highlighted within these pathways for clarity. Red in the path map denotes the up-regulated metabolites, blue denotes the down-regulated metabolites, whereas white denotes the metabolites that were not present or were not detected in the species. Each pathway exhibited a statistically significant degree of enrichment. (*P* < 0.05; Fig. S2) (Appendix).

### Correlation analysis of the effects of HFD on gut microbiota and fecal metabolic phenotypes of CIA mice

To determine the correlations between the relative abundance of microorganisms and metabolite reaction intensity data, the Pearson correlation algorithm was utilized. The Pearson correlation coefficient values were used to construct heat map and matrix diagram.

The heat map showing the Spearman correlation coefficients between fecal metabolite concentrations and relative abundance of individual genera. Color intensity denotes the correlation degree.

The correlation matrix of the TOP20 correlation analysis results was drawn, with each row representing different metabolites and each column denoting the microorganism. In the figure, the color depth is proportional to correlation. Circle size demotes the size of the correlation. The larger the circle, the greater the correlation.

**Fig. 11:**
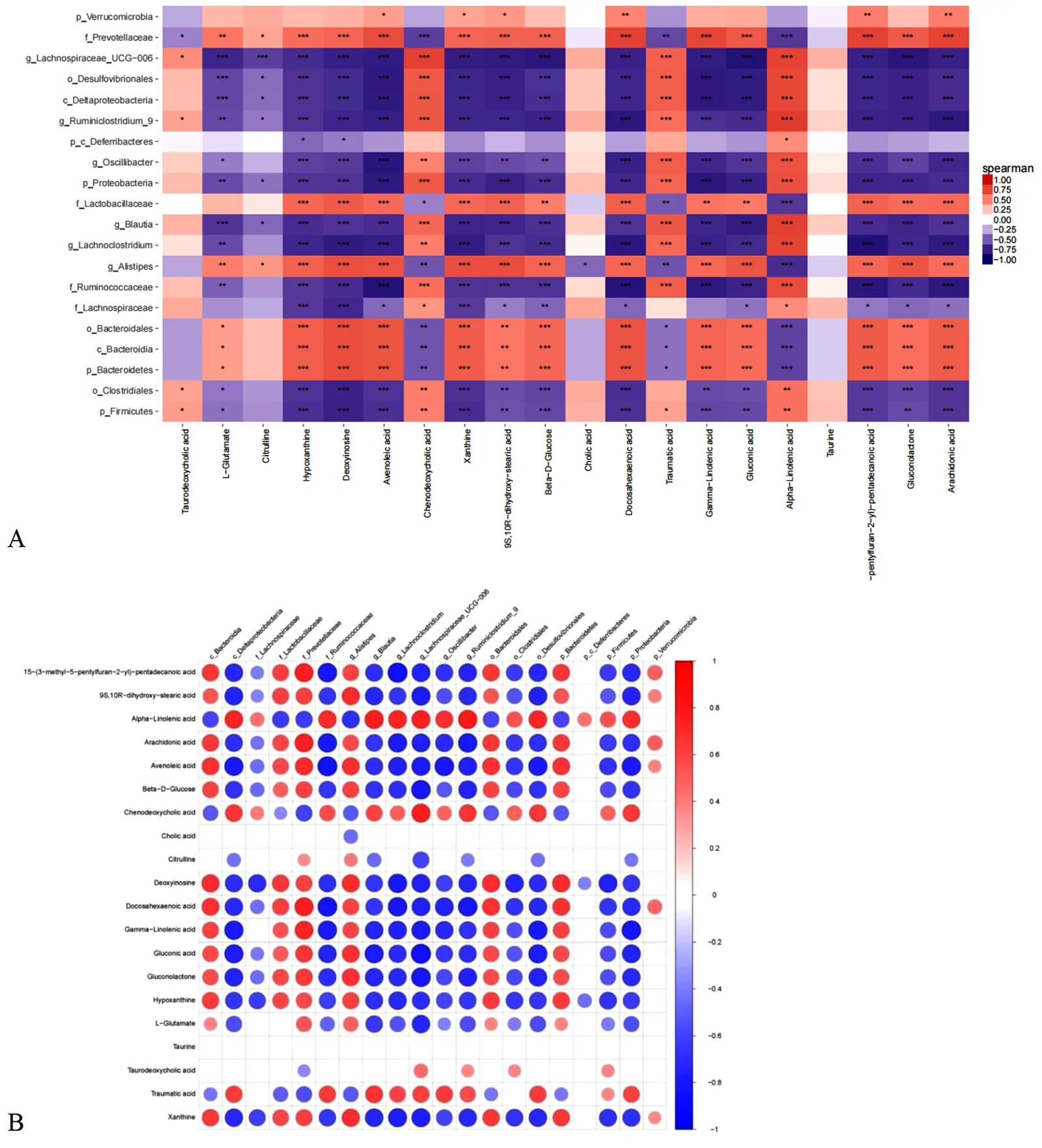
Correlation analysis of the effects of HFD on gut microbiota and fecal metabolic phenotypes of CIA mice. (**A**) The heat-map: Correlations between gut microbiota genera and altered fecal metabolites. (**B**) Correlation matrix drawing of TOP20 results of correlation analysis between differential species/OTU and differential metabolites. n = 8 per group. Significant differences were indicated as *P < 0.05, **P < 0.01, ***P < 0.001

Spearman correlation analysis revealed the correlations between the bacterial community and metabolites (Table 1) (Fig. 11A, B).

**Table 1:** Correlation analysis of the effects of HFD on gut microbiota and fecal metabolic phenotypes of CIA mice

Bacteria community	metabolites	r	*P*-value
*p_Verrucomicrobia*	Avenoleic acid	0.37	*P* < 0.001
	Xanthine	0.36	*P* < 0.001
	9S,10 R-dihydroxy-stearic acid	0.40	*P* < 0.001
	Docosahexaenoic acid	0.47	*P* < 0.001
	15-(3-methyl-5-pentylfuran-2-yl)-pentadecanoic acid	0.50	*P* < 0.001
	Arachidonic acid	0.50	*P* < 0.001
*f_Prevotellaceae*	L-Glutamate	0.53	*P* < 0.01
	Citrulline	0.35	*P* < 0.05
	Hypoxanthine	0.57	*P* < 0.001
	Deoxyinosine	0.60	*P* < 0.001
	Avenoleic acid	0.70	*P* < 0.001
	Xanthine	0.61	*P* < 0.001
	9S,10 R-dihydroxy-stearic acid	0.61	*P* < 0.001
	Beta-D-Glucose	0.61	*P* < 0.001
	Docosahexaenoic acid	0.76	*P* < 0.001
	Gamma-Linolenic acid	0.74	*P* < 0.001
	Gluconic acid	0.66	*P* < 0.001
	15-(3-methyl-5-pentylfuran-2-yl)-pentadecanoic acid	0.76	*P* < 0.001
	Gluconolactone	0.65	*P* < 0.001
	Arachidonic acid	0.75	*P* < 0.001
*g_Lachnospiraceae_UCG-006*	Taurodeoxycholic acid	0.45	*P* < 0.05
	Chenodeoxycholic acid	0.76	*P* < 0.001
	Traumatic acid	0.62	*P* < 0.001
	Alpha-Linolenic acid	0.75	*P* < 0.001
*o_Desulfovibrionales*	Chenodeoxycholic acid	0.65	*P* < 0.001
	Traumatic acid	0.63	*P* < 0.001
	Alpha-Linolenic acid	0.73	*P* < 0.001
*c_Deltaproteobacteria*	Chenodeoxycholic acid	0.66	*P* < 0.001
	Traumatic acid	0.63	*P* < 0.001
	Alpha-Linolenic acid	0.73	*P* < 0.001
*g_Ruminiclostridium_9*	Taurodeoxycholic acid	0.36	*P* < 0.05
	Chenodeoxycholic acid	0.66	*P* < 0.001
	Traumatic acid	0.56	*P* < 0.001
	Alpha-Linolenic acid	0.79	*P* < 0.001
p*_*c*_*Deferribacteres	Alpha-Linolenic acid	0.44	*P* < 0.05
*g_Oscillibacter*	Chenodeoxycholic acid	0.48	*P* < 0.01
	Traumatic acid	0.64	*P* < 0.001
	Alpha-Linolenic acid	0.68	*P* < 0.001
*p_Proteobacteria*	Chenodeoxycholic acid	0.65,	*P* < 0.001
	Traumatic acid	0.60	*P* < 0.001
	Alpha-Linolenic acid	0.69	*P* < 0.001
*f_Lactobacillaceae*	Hypoxanthine	0.59	*P* < 0.001
	Deoxyinosine	0.65	*P* < 0.001
	Avenoleic acid	0.58	*P* < 0.001
	Xanthine	0.59	*P* < 0.001
	9S,10 R-dihydroxy-stearic acid	0.61	*P* < 0.001
	Beta-D-Glucose	0.50	*P* < 0.01
	Docosahexaenoic acid	0.62	*P* < 0.001
	Gamma-Linolenic acid	0.54	*P* < 0.01
	Gluconic acid	0.52	*P* < 0.01
	15-(3-methyl-5-pentylfuran-2-yl)-pentadecanoic acid	0.61	*P* < 0.001
	Gluconolactone	0.58	*P* < 0.001
	Arachidonic acid	0.59	*P* < 0.001
*g_Blautia*	Chenodeoxycholic acid	0.60	*P* < 0.001
	Traumatic acid	0.67	*P* < 0.001
	Alpha-Linolenic acid	0.77	*P* < 0.001
*g_Lachnoclostridium*	Chenodeoxycholic acid	0.50	*P* < 0.01
	Traumatic acid	0.59	*P* < 0.001
	Alpha-Linolenic acid	0.74	*P* < 0.001
*g_Alistipes*	L-Glutamate	0.50	*P* < 0.01
	Citrulline	0.41	*P* < 0.05
	Hypoxanthine	0.62	*P* < 0.001
	Deoxyinosine	0.69	*P* < 0.001
	Avenoleic acid	0.68	*P* < 0.001
	Xanthine	0.70	*P* < 0.001
	9S,10 R-dihydroxy-stearic acid	0.70	*P* < 0.001
	Beta-D-Glucose	0.61	*P* < 0.001
	Docosahexaenoic acid	0.60	*P* < 0.001
	Gamma-Linolenic acid	0.59	*P* < 0.001
	Gluconic acid	0.67	*P* < 0.001
	15-(3-methyl-5-pentylfuran-2-yl)-pentadecanoic acid	0.59	*P* < 0.001
	Gluconolactone	0.68	*P* < 0.001
	Arachidonic acid	0.57	*P* < 0.001
*f_Ruminococcaceae*	Chenodeoxycholic acid	0.56	*P* < 0.01
	Traumatic acid	0.63	*P* < 0.001
	Alpha-Linolenic acid	0.71	*P* < 0.001
*f_Lachnospiraceae*	Chenodeoxycholic acid	0.42	*P* < 0.05
	Alpha-Linolenic acid	0.44	*P* < 0.05
*o_Bacteroidales\p_Bacteroidia\o_Bacteroidetes*	L-Glutamate	0.38	*P* < 0.05
	Hypoxanthine	0.64	*P* < 0.001
	Deoxyinosine	0.70	*P* < 0.001
	Avenoleic acid	0.68	*P* < 0.001
	Xanthine	0.64	*P* < 0.001
	9S,10 R-dihydroxy-stearic acid	0.53	*P* < 0.01
	Beta-D-Glucose	0.59	*P* < 0.001
	Docosahexaenoic acid	0.68	*P* < 0.001
	Gamma-Linolenic acid	0.60	*P* < 0.001
	Gluconic acid	0.58	*P* < 0.001
	15-(3-methyl-5-pentylfuran-2-yl)-pentadecanoic acid	0.65	*P* < 0.001
	Gluconolactone	0.56	*P* < 0.001
	Arachidonic acid	0.64	*P* < 0.001
*o_Clostridiales*	Taurodeoxycholic acid	0.37	*P* < 0.05
	Chenodeoxycholic acid	0.50	*P* < 0.01
	Alpha-Linolenic acid	0.55	*P* < 0.01
*p_Firmicutes*	Taurodeoxycholic acid	0.39	*P* < 0.05
	Chenodeoxycholic acid	0.50	*P* < 0.01
	Traumatic acid	0.37	*P* < 0.05
	Alpha-Linolenic acid	0.55	*P* < 0.01

### HFD alters the association between inflammatory cytokines, blood lipid parameters and core gut microbiota

Spearman correlation analysis was performed to determine the correlation between TC, TG, inflammatory Cytokines and the gut microbiome.

**Fig. 12:**
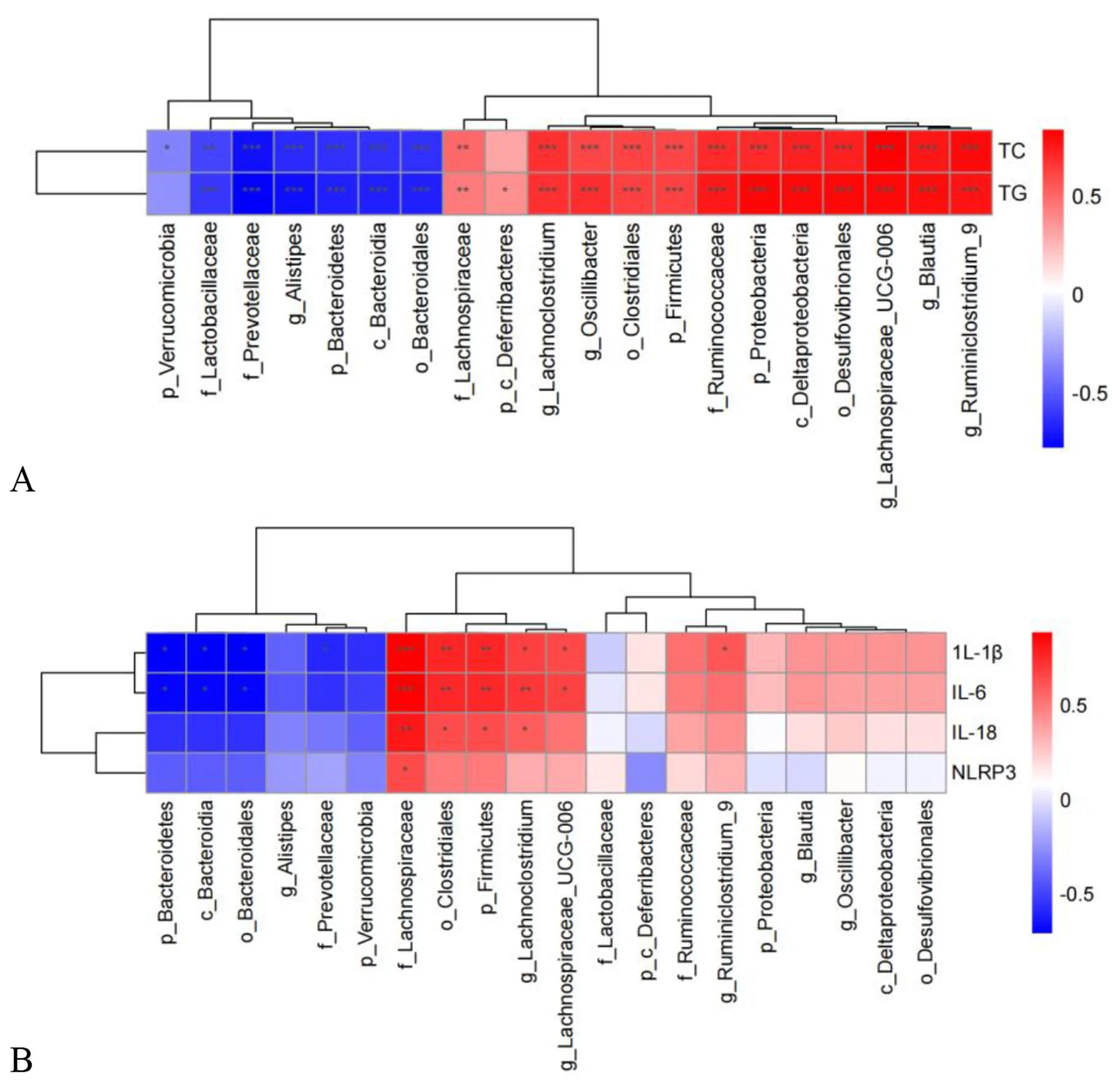
Correlations between inflammatory cytokines and abundance of core gut microbiota in the four groups. (**A**) Correlations between TC, TG and gut microbiota. (**B**) Correlations between inflammatory factors and gut microbiota. n = 8 per group. * * *P < 0.001, * *P < 0.01, and * *P* < 0.05

In the CT group, the increase in abundance of *Lachnospiraceae*_UCG_006 (0.84)(0.81), *Oscillibacter* (0.61)(0.69), *Proteobacteria* (0.70)(0.82), *Firmicutes* (0.62)(0.63), *Ruminiclostridium*-9 (0.80) (0.77) and *Blautia* (0.77)(0.80) were positively correlated with TC and TG levels (*P* < 0.001). The reduction in abundance of *Verucomicrobia* (−0.36) (−0.30) (*P* < 0.05), *Alisitipes* (−0.64) (−0.72) (*P* < 0.001), and *Bacteroidetes* (−0.61) (−0.66) (*P* < 0.001) was negatively correlated with TC and TG levels (Fig. 12A).

In the CT group, the increased abundance of *Lachnospiraceae* was positively correlated with IL-1β(0.89) (*P* < 0.001), IL-6(0.88) (*P* < 0.001), NLRP3 (0.66) (*P* < 0.05), and IL-18 (0.81) (*P* < 0.01) levels whereas increased abundance of *Clostridiales* was positively correlated with IL-1β (0.77) (*P* < 0.01), IL-6(0.76) (*P* < 0.01), and IL-18 levels (0.65) (*P* < 0.05); whereas the increased abundance of *Firmicutes* was positively correlated with IL-1β (0.77) (*P* < 0.01), IL-6(0.76) (*P* < 0.01), and IL-18 (0.65) levels (*P* < 0.05). The increase in abundance of *Lachnoclostridium* was positively correlated with IL-1β (0.62) (*P* < 0.05), IL-6(0.72) (*P* < 0.01); IL-18 (0.60) levels (*P* < 0.05); while the increase in abundance of *Ruminiclostridium_9* was positively correlated with IL-1β (0.62) levels (*P* < 0.05) (Fig. 12B).

## Discussion

HFD can lead to hyperlipidemia and obesity, leaving the body in a state of chronic inflammation, accompanied by changes in its flora [86, 87]. However, it has not been determined whether the HFD-induced chronic inflammatory state has an effect on RA, and whether its role is related to gut microbiota regulation. To verify this result, we used obese CIA DBA/1 mice models. We observed that weight and other obesity parameters (TC and TG levels) of mice fed on HFD for 14 weeks were significantly increased, and that obese CIA mice had higher arthritis index and histological scores. Our findings are consistent with those of some previous studies [88, 89]. Pro-inflammatory cytokines (IL-6, IL-1β, NLRP3 and IL-18) are key functional molecules contributing to RA occurrence and development [42, 77, 90–97]. In this study, we found that HFD increased the expression levels of inflammatory cytokines (IL-6, IL-18, NLRP3 and IL-1β) and aggravated inflammatory responses in CIA mice. However, the mechanism underlying these effects need to be determined in future studies.

The gut microbiome is an important environmental factor in RA occurrence [98], as it plays an important role in metabolic homeostasis [99, 100]. To establish the mechanisms via which HFD aggravate CIA inflammatory responses, we performed integrated 16S rRNA gene sequencing and metabolomics based on LC-MS to assess the effects of HFD on gut microbiota and fecal metabolic phenotypes of CIA mice. The abundance of gut microbial communities at the phylum and genus levels varied significantly among groups. The metabolic profiles were also significantly different among the groups. These findings demonstrate that HFD may influence the occurrence of RA by affecting gut microbiota.

### 16s rRNA sequencing

Furthermore, 16S rRNA sequencing was conducted to characterize gut microbiota of DBA1 mice with arthritis following administration of collagen. Results showed that the difference in microbiota distribution after induction was significant, and the abnormal microbiota features were observed in the T group. As shown in Fig. 4C, D, the Chao index, and PCoA of α-diversity all showed that the four groups showed significant differences in the microbiota, suggesting that CIA and HFD were independent intervention factors. We found that a HFD led to major changes in gut microbiome composition of CIA mice models. Moreover, these changes in bacterial abundance may be the reason for the aggravated inflammation in the CT group and the role of some bacteria was revealed.

The increased abundance of *Lactobacillus* and *Bacteroides* can positively regulate obesity [101, 102]. Moreover, some *Lactobacillus* species can induce arthritis [103]. In this study, the abundance of *Lactobacillus* in group C was significantly higher compared with that of the other groups. In animal models, similarity analysis revealed differences in gut microbiota structures between CIA susceptible and resistant mice before arthritis onset. Before the onset of arthritis, gut abundance of *Desulfovibrionaceae* and *Lachnospiraceae* bacteria in CIA susceptible mice were significantly less than those of CIA-resistant mice, while the abundance of Lactobacillus in the intestines of CIA susceptible mice was high relative to that in resistant mice. After arthritis onset, gut abundance of *Bacteroidaceae, Lachnospiraceae* and s24-7 families in CIA susceptible mice was significantly increased, compared to before the onset [31]. In human studies, abundance of *Clostridium asparagiforme, Lachnospiraceae, Lactobacillus* sp. and *Ruminococcus lactaris* in the intestines of RA patients were found to be significantly increased. *Clostridium asparagiforme* and *Bacteroides sp* (*Bacteroides*) were positively correlated with immunoglobulin A antibody (IgA), and abnormal arginine transport and metabolism in the microbial environment were noted [11]. In a recent study, 16S rRNA gene sequencing methods confirmed significant changes in gut microbiota composition in RA patients compared with healthy individuals. This was revealed by the increased levels of *Bacteroidetes* and *Proteobacteria* phyla [104]. The abundance of *Lachnospiraceae, Bacteroidaceae*, and S24-7 were markedly increased in CIA susceptible mice, which contributed to the increase in taxonomic diversity as CIA progressed. The S24-7 and *Bacteriodales* can prevent RA, while *Lachnospiraceae* has the opposite effect [105]. *Verrucobacteria* can also positively regulate obesity [106]. The *Lachnospiraceae* bacterium A4 has been shown to regulate T cell balance [107]. *Desulfovibrio* and *Alistipes* are associated with gut mucosal thickness [108].

These studies highlight the primary bacterial genera implicated in mitigating obesity-related RA. Future investigations should delve into the principal pathways by which microorganisms influence RA. Currently, the majority of studies concentrate on multigroup analyses of the microbiome and metabolomics. Metabolites originating from the intestinal microflora have been found to possess immunomodulatory properties that regulate the differentiation and function of immune cells [109, 110]. Some studies have also shown that the imbalance in intestinal microflora occurs in the early clinical stage of RA and is closely related to the occurrence of arthritis, including molecular mimicry, microbiome derived metabolites, impairment of intestinal barrier function, and microbiome induced intestinal immune response. The imbalance of intestinal microflora is connected to the progression of RA through these pathways [111, 112]. Similarly, it has been suggested that the mechanism underlying the imbalance of gut microbiota leading to RA may involve the regulation of immune function by metabolites produced by gut microbiota [10, 113]. Therefore, in future, we will explore the changes in gut microbiota metabolites caused by a HFD, and further analyze its mechanism of action on RA.

### Pathway analysis of fecal metabolomics

Non-targeted fecal metabolomics studies have been used to reveal metabolic phenotypic changes that are associated with gut microbiome interference in disease development.

In this study, the HFD was given to CIA mice, the metabolic profiles of each mice group were significantly different. There were 22 highly expressed fecal metabolites in the HFD fed CIA composite model group. As shown in Fig. 9A, the identified biomarkers showed that HFD aggravated the inflammatory response of CIA mice and oleic acid was the main biomarker.

Further stratified analysis of all metabolite classes showed that long-chain fatty acids in the high-fat group changed significantly. Traumatic acid, α-linolenic acid, linolenic acid was highest in the CT group. These gut microbiota-associated metabolites play vital roles in RA-induced metabolic changes.

A reduction in dietary linoleic acid/alpha-linolenic acid ratio reduced cartilage damage in modulator-induced arthritis rat models [114]. In addition, fibroblast-like synoviocytes (FLS) oleic acid and linoleic acid levels in RA patients were much higher than those in OA patients [115]. When several common fatty acids (FAs) (e.g., stearic acid, oleic acid, eicosapentaenoic acid, and linoleic acid) were used to stimulate human RA synovial fibroblasts, secretion of inflammatory factors such as IL-6 and chemokines was increased [116, 117]. In vitro studies of normal chondrocytes exposed to different FA under inflammatory conditions showed that palmitic acid has pro-apoptotic and pro-inflammatory roles [118]. There could be increased sensitivity to palmitic acid and linoleic acid in RA compared with OA osteoclasts [119]. Arachidonic acid causes imbalances between proliferation and apoptosis of FLS and promotes Pannus formation [120, 121]. Therefore, we postulated that metabolites such as fatty acids are biomarkers of HFD-induced RA exacerbation. High-quality clinical studies should be performed to confirm this outcome.

Enrichment analysis of differentially expressed metabolites showed that the enriched metabolic pathways were primary bile acid biosynthesis, arginine biosynthesis, sphingolipid metabolism, purine metabolism, linoleic acid metabolism, oxytocin signaling pathway, aminoacyl-tRNA biosynthesis, pentose phosphate pathway and the sphingolipid signaling pathway. Next, we will analyze the associated metabolic pathways and key targets.

#### Primary bile acid biosynthesis

Primary bile acid biosynthesis is a distinct metabolic pathway for taurodeoxycholic acid (TDCA), taurine, and deoxycholic acid (DCA). Bile acid (BA) is a product of cholesterol metabolism and clearance. Cholesterol accumulation promotes inflammatory responses, inflammasome activation, and the production of monocytes and neutrophils. Differentially expressed metabolites in this study included TDCA, taurine, DCA, and cholic acid (CA), which are metabolized by gut bacteria [122].

#### Arginine biosynthesis

Arginine biosynthesis is another metabolic pathway that was found to be significantly enriched. However, the role of arginine metabolism in RA has not been fully confirmed. In evolutionary processes, the amino acid metabolism pathway is the key checkpoint of adaptive immune inflammatory responses [123, 124]. Arg is a conditionally essential basic amino acid [125]. The NOS enzymes convert Arg to nitric oxide (NO) and citrulline, while arginase catalyzes the conversion of Arg to ornithine (Orn) and urea [126]. These immunomodulatory effects are characterized by rate-limiting enzymes and depend on production of biologically active metabolites of specific amino acids in the microenvironment. Expression of each degradation pathway are usually strictly regulated [127]. Changes in gut microbiome composition have been observed in RA patients, and host’s “co-metabolism” of amino acids is also affected [128, 129]. Dysfunction in the metabolism of arginine may contribute to RA because increased arginase activity can reduce the production of vasodilator NO [130, 131].

#### Purine metabolism

Patients with RA exhibit elevated uric acid levels and excess deposits of uric acid in joints, soft tissues, and cartilage [132, 133]. Inosine and hypoxanthine are intermediates and derivatives of purine degradation [134]. In this study, hypoxanthine, xanthine and inosine were detected as differentially expressed biomarkers in fecal samples, indicating abnormal purine metabolism in HFD-CIA models. The implication of oxidative stress in the pathogenesis of RA may arise from the ability of uric acid to neutralize ROS and exert an anti-inflammatory effect [135, 136].

#### Linoleic acid and fatty acid metabolism

Linoleic acid metabolism is a subset of fatty acid metabolism known to generate energy and serve as a crucial source for various lipid substances [137]. In this study, we identified several fatty acid metabolites, including palmitoleic acid, oleic acid, arachidonic acid, and linoleic acid. Unsaturated fatty acids, such as these, have been shown to stimulate the production of inflammatory cytokines, thus contributing to the onset and progression of RA [138, 139]. Linoleic acid (LA) is a precursor of n-6 PUFA arachidonic acid (AA) [140], which produces a series of eicosanic acids that have pro-inflammatory and pro-thrombotic effects [141, 142].

Physiologically, LA has been shown to regulate blood lipid levels, reduce the levels of low-density lipoprotein (LDL), cholesterol, triglyceride as well as ApoB, and increase the levels of high-density lipoprotein and cholesterol [143, 144]. AA is a potential proinflammatory leukotriene precursor and is considered to be a typical proinflammatory fatty acid, however, there is increasing evidence that its roles may be more complex [145].

#### Pentose phosphate pathway

In RA CD4 T cells, glucose is redirected from glycolysis towards the pentose phosphate pathway (PPP). Transcriptional suppression of phosphofructokinase/fructose diphosphatase 3 (PFKFB3) results in reduced ATP and pyruvate production. Conversely, increased expression of glucose-6-phosphate dehydrogenase (G6PD) facilitates glucose entry into the PPP [146–152]. Consequently, adipogenesis may facilitate the tissue invasiveness of RA T cells. The CD4 T cells in RA patients were shown to change the distribution of glucose to different pathways, shunting glucose to PPP. Therefore, PPP may be one of the metabolic pathways via which the HFD aggravated inflammatory responses in CIA mice models.

### Correlations between gut microbiota and fecal metabolites

The Pearson correlation analysis revealed significant associations between gut microbiota and fecal metabolites.

There was a specific, significant correlation between different gut microbiota and fecal metabolites. Lachnospiraceae_UCG-006, *Desulfovibrionales, Deltaproteobacteria, Ruminiclostridium*_9, *Oscillibacter, Blautia, Clostridiales* and Firmicutes were the dominant species in the CT group, and were highly correlated with taurodeoxycholic acid, chenodeoxycholic acid, traumatic acid, and alpha-linolenic acid, indicating that HFD aggravate CIA by altering gut microbiota abundance and regulating primary bile acid biosynthesis via the linoleic acid metabolism pathway.

The expression level of *Verrucomicrobia* and *Bacteroidales* was lower in the CT group, but were strongly correlated with avenoleic acid, xanthine, 9S, 10R-dihydroxy-stearic acid, docosahexaenoic acid, 15-(3-methyl-5-pentylfuran-2-yl)-pentadecanoic acid, and arachidonic acid, indicating that the microbes were associated with purine and fatty acid metabolism.

The concentration of Alistipes was low in the CT group, and was strongly correlated with deoxyinosine, avenoleic acid, xanthine, 9S, 10R-dihydroxy-stearic acid, Beta-D-Glucose, docosahexaenoic acid, gamma-linolenic acid, gluconic acid, 15-(3-methyl-5-pentylfuran-2-yl)-pentadecanoic acid, gluconolactone, and arachidonic acid. These findings suggest that purine metabolism, linoleic acid metabolism, and pentose phosphate pathway are highly activated during RA exacerbation, which may be closely related to the functions of gut microbiota. *Blautia, Coprococcus* and *Ruminococcaceae* were the dominant bacteria in the CT group. It has been shown that DCA is produced by the catalytic actions of 7α-dehydroxylase, which is present in bacteria, including Eubacterium and *Clostridiumgenus* [153]. *Ruminococcaceae and Blautia* are known to produce 7α-dehydroxylase, resulting in increased fecal DCA levels [154]. Hong Lin reported that a HFD resulted in a slight increase in total bile acid pool, particularly plasma and liver tissue deoxycholic acid (DCA) and taurodeoxycholic (TDCA) levels, consistent with increased DCA levels. These changes were associated with increased abundance of *Blautia, Coprococcus, Intestinimonas, Lactococcus, Roseburia, and Ruminococcus* [155].

We assessed the correlations between changes in gut microbiota and TC, TG and inflammatory factors in the four groups. There were positive correlations between *Lachnospiraceae, Firmicutes, Lachnoclostridium, Ruminiclostridium_9* and IL-1β, IL-6, NLRP3, and IL-18. These results showed that changes in gut microbiota abundance were significantly correlated with the level of fecal metabolites and inflammatory factors, implying that HFD not only altered the gut microbiota of CIA mice, but also affected the host metabolic phenotype, disrupting the host metabolite homeostasis and aggravating inflammatory responses. This is the first study to assess the effects of HFD on RA in CIA mice models. However, this study has several limitations: i. We only used the LC-MS method for detection, which may be ignored for small molecules with m/z values below 500, making it difficult to fully present the entire metabolic processes. In future, we will use the GC-MS technology to fully reveal the changes of metabolites *in vivo* and to verify the influence of HFD on RA. ii. In this preliminary study, we found an association between gut microbiome and fecal metabolites. Future studies should incorporate specific antibiotic interventions to manipulate gut microbiome composition. In addition, considering the use of mouse models in our research, it is important to acknowledge potential differences in gut microbiota between humans and mice. Thus, collecting human samples from obese RA patients for gut microbiota and metabolomics analyses could unveil HFD-associated biomarkers linked to RA exacerbation.

## Conclusions

The HFD aggravated the arthritis status of CIA mice, and significantly altered their gut microbial composition as well as fecal metabolic phenotypes. Some of the altered gut microbiota genera were strongly correlated with changes in levels of fecal metabolites, TC, TG and inflammatory cytokines. The HFD increased the abundance of *Firmicutes, Proteobacteria, Blautia, Oscillibacter, Ruminiclostridium-9, lachnospiraceae_UCG 006* and decreased the abundance of *Bacteroidetes, Verrucomicrobia and Alistipes*. Moreover, the HFD altered the dynamic balance in the host’s metabolism by affecting primary bile acid biosynthesis, arginine biosynthesis, sphingolipid metabolism, purine metabolism, linoleic acid metabolism, oxytocin signaling pathway, aminoacyl-tRNA biosynthesis, pentose phosphate pathway, sphingolipid signaling pathway, and aggravating the inflammatory reactions. In summary, gut microbiome-related metabolites can be used as biomarkers of the effects of HFD on CIA mice, and can exploited to develop microbiome-based RA diagnosis, prevention, and treatment modalities.

## Electronic supplementary material

Below is the link to the electronic supplementary material.

Supplementary Material 1

## Data Availability

All data supporting the conclusions in this study are included in the Supplementary Materials. The datasets analyzed during the current study are available from the corresponding author upon reasonable request.
