# Supplementary material for: High fat diet increases the severity of collagen-induced arthritis in mice by altering the gut microbial community
Source: Adv Rheumatol. 2024 May 30;64:44. doi: 10.1186/s42358-024-00382-y (PMC13488716; doi:10.1186/s42358-024-00382-y)
Supplement: Supplementary file 1 [file 42358_2024_382_MOESM1_ESM.doc]

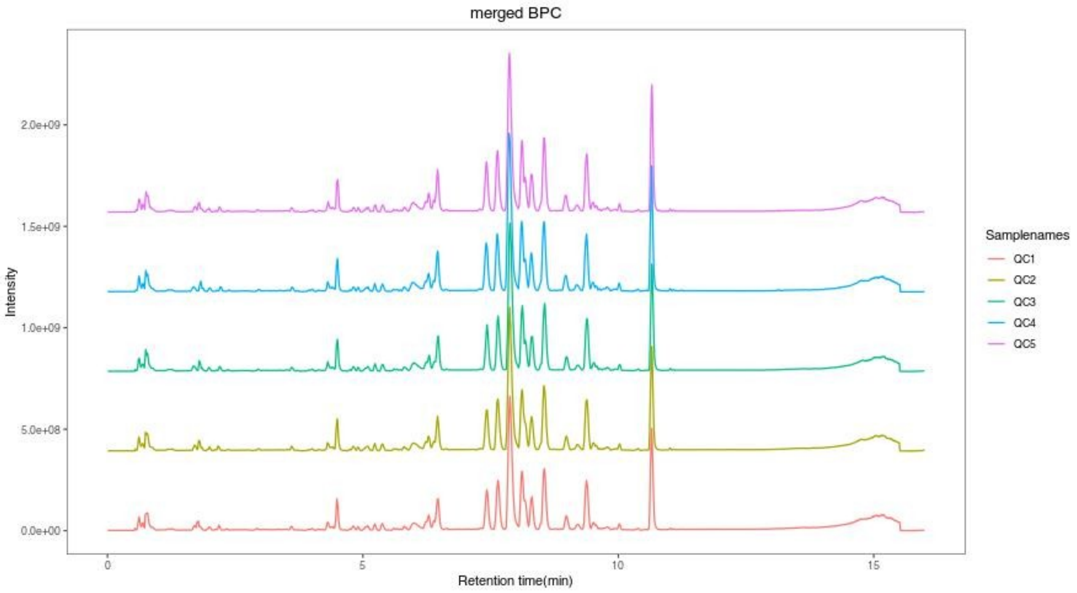
merged BPC
Intensity
2.0e+09
1.5e+09
1.0e+09
5.0e+08
0.0e+00
0
5
10
15
Retention time(min)
Samplenames
QC1
QC2
QC3
QC4
QC5

A

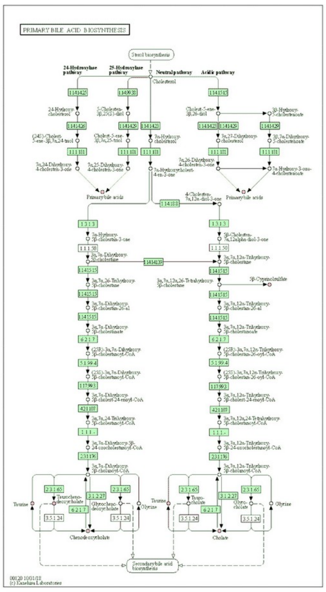
PRIMARY BILE ACID BIOSYNTHESIS

B

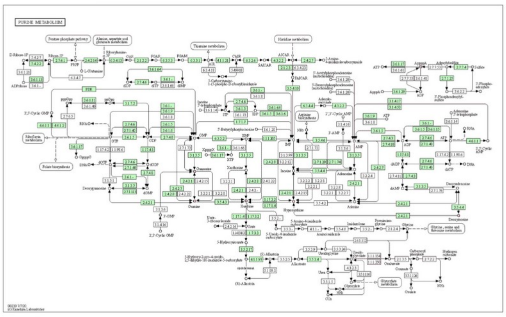

C

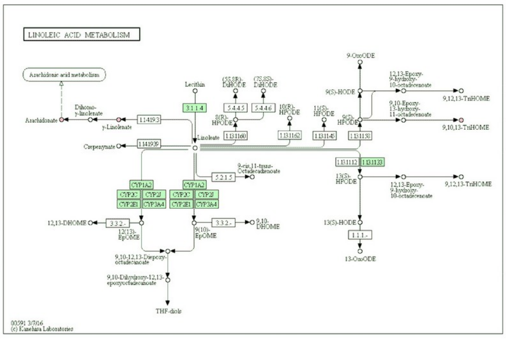
LINOLEIC ACID METABOLISM

D

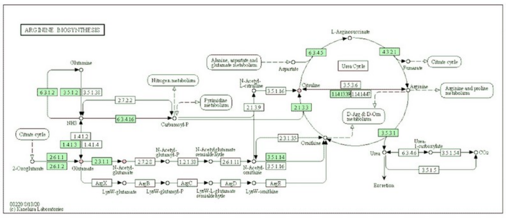
ARGININE BIOSYNTHESIS

E

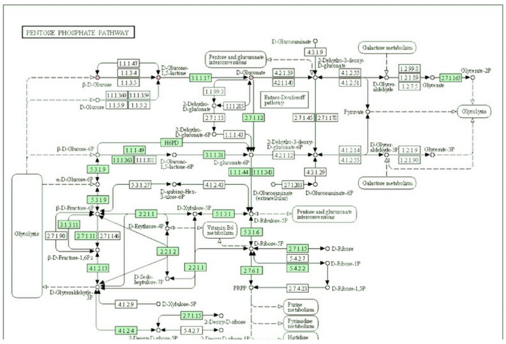
PENTOSE PHOSPHATE PATHWAY
